# Seasonal Phenology and Species Composition of the Aphid Fauna in a Northern Crop Production Area

**DOI:** 10.1371/journal.pone.0071030

**Published:** 2013-08-13

**Authors:** Sascha M. Kirchner, Lea Hiltunen, Thomas F. Döring, Elina Virtanen, Jukka P. Palohuhta, Jari P. T. Valkonen

**Affiliations:** 1 Department of Agricultural Sciences, University of Helsinki, Helsinki, Finland; 2 MTT AgriFood Research Finland, Oulu, Finland; 3 Department of Crop and Animal Sciences, Humboldt-Universität, Berlin, Germany; 4 Finnish Seed Potato Centre, Tyrnävä, Finland; University of Miami, United States of America

## Abstract

**Background:**

The species diversity of aphids and seasonal timing of their flight activity can have significant impacts on crop production, as aphid species differ in their ability to transmit plant viruses and flight timing affects virus epidemiology. The aim of the study was to characterise the species composition and phenology of aphid fauna in Finland in one of the northernmost intensive crop production areas of the world (latitude 64°).

**Methodology/Principal Findings:**

Flight activity was monitored in four growing seasons (2007–010) using yellow pan traps (YPTs) placed in 4–8 seed potato fields and a Rothamsted suction trap. A total of 58,528 winged aphids were obtained, identified to 83 taxa based on morphology, and 34 species were additionally characterised by DNA barcoding. Seasonal flight activity patterns analysed based on YPT catch fell into three main phenology clusters. Monoecious taxa showed early or middle-season flight activity and belonged to species living on shrubs/trees or herbaceous plants, respectively. Heteroecious taxa occurred over the entire potato growing season (ca. 90 days). Abundance of aphids followed a clear 3-year cycle based on suction trap data covering a decade. *Rhopalosiphum padi* occurring at the end of the potato growing season was the most abundant species. The flight activity of *Aphis fabae*, the main vector of *Potato virus Y* in the region, and *Aphis gossypii* peaked in the beginning of potato growing season.

**Conclusions/Significance:**

Detailed information was obtained on phenology of a large number aphid species, of which many are agriculturally important pests acting as vectors of plant viruses. *Aphis gossypii* is known as a pest in greenhouses, but our study shows that it occurs also in the field, even far in the north. The novel information on aphid phenology and ecology has wide implications for prospective pest management, particularly in light of climate change.

## Introduction

The life cycles of aphid species differ and exhibit various patterns of polymorphism [1]. Aphids can be grouped according to their relationships with host plants to heteroecious (host-alternating) and monoecious (non host-alternating) species [2], which is reflected in the phenology of aphid populations, i.e., in the specific temporal patterns of flight activities within a year, or, as in agricultural contexts, within a growing season. The seasonal pattern of flight activity of host-alternating species can be seen as an indicator of the optimal time for host transfer between primary (mainly woody) and secondary (herbaceous) hosts. In non host-alternating species, seasonal flight patterns are likely to reflect the time window for spreading clones before host quality starts to deteriorate [2].

Aphid phenology, however, is not only dependent on species-specific relationships between the aphid and the host plant, but it is also largely influenced by local environmental factors such as photoperiod and mean winter temperatures [3]. During the growing season, climatic factors influencing aphid phenology vary and are challenging to predict. Further complexities are introduced, because the timing of trap catch is not only dependent on innate phenology but also aphid abundance. High abundance will lead to increased probability of capture and earlier recognition of the flight. Aphid abundance is particularly difficult to predict, due to the dynamic responses of aphid populations to predation, diseases or parasitation [4]. For these reasons, regularities in the flight activities of aphids are notoriously difficult to extract even from long term data sets.

Phenology can have a significant impact on the pest status of aphid species. For example, phenology determines at which growth stage the crop is likely to be invaded by aphids and which crops are likely to be affected most severely [5]. Aphid phenology has particularly important implications to transmission of aphid-vectored plant viruses, because inoculation of plants with viruses at an early growth stage results in higher rates of infection [6]–[9]. Therefore, improving the knowledge on aphid phenology is useful for planning plant protection strategies, in particular with regards to aphid-transmitted viruses.

At a broad geographic scale, comparing aphid species occurring in temperate zones with those found in subarctic regions reveals that the higher latitudes are characterised by a lower proportion of anholocyclic clones and winged (alate) aphids [10]. The flights of aphids in the spring start later [11] consistent with the late beginning of plant growing season. Hence the life cycle of aphids is limited to a short time window, which reduces the time, e.g., of coexistence of viruses and vectors and reduces infection pressure [9].

The Tyrnävä-Liminka area of Finland (ca. 64°N) is characterised by a short thermal growing season from May to early October, warm summers and relatively cold winters. The region is one of the five European High Grade Seed Potato Production Zones (HG zones) recognised by the European Union. In 2012, ca. 27300 tonnes of seed potatoes were produced on 1000 ha of field [12]. Systematic studies on aphid flight phenology in Tyrnävä-Liminka area and other northern agricultural regions in the world are relatively rare. The studies have been focused on selected aphid species, such as cereal aphids transmitting barley yellow dwarf viruses or the relatively few species transmitting *Potato virus Y* (PVY) in Finland and Sweden [13]–[16]. Therefore, the aim of this study was to provide more comprehensive and systematic information on the phenology and species composition of the aphid fauna in the Tyrnävä-Liminka area representing an agriculturally important region with an unusually northern location. The data have been utilized in a novel modelling approach revealing that *Aphis fabae* is the main vector of *Potato virus Y* in the region [9], but the phenology and species composition of the aphids are reported here. The low probability of anholocycly [17] in the region provided unique opportunities to study aphid phenology in a less complexity-laden environment where extraction of phenological patterns may be easier.

## Materials and Methods

### Ethics Statement

No specific permits were required for the described field study. Additionally, we got oral approvals for insect sampling from all private land owners. The collections did not involve endangered or protected species.

### Environmental conditions

**Figure 1:**
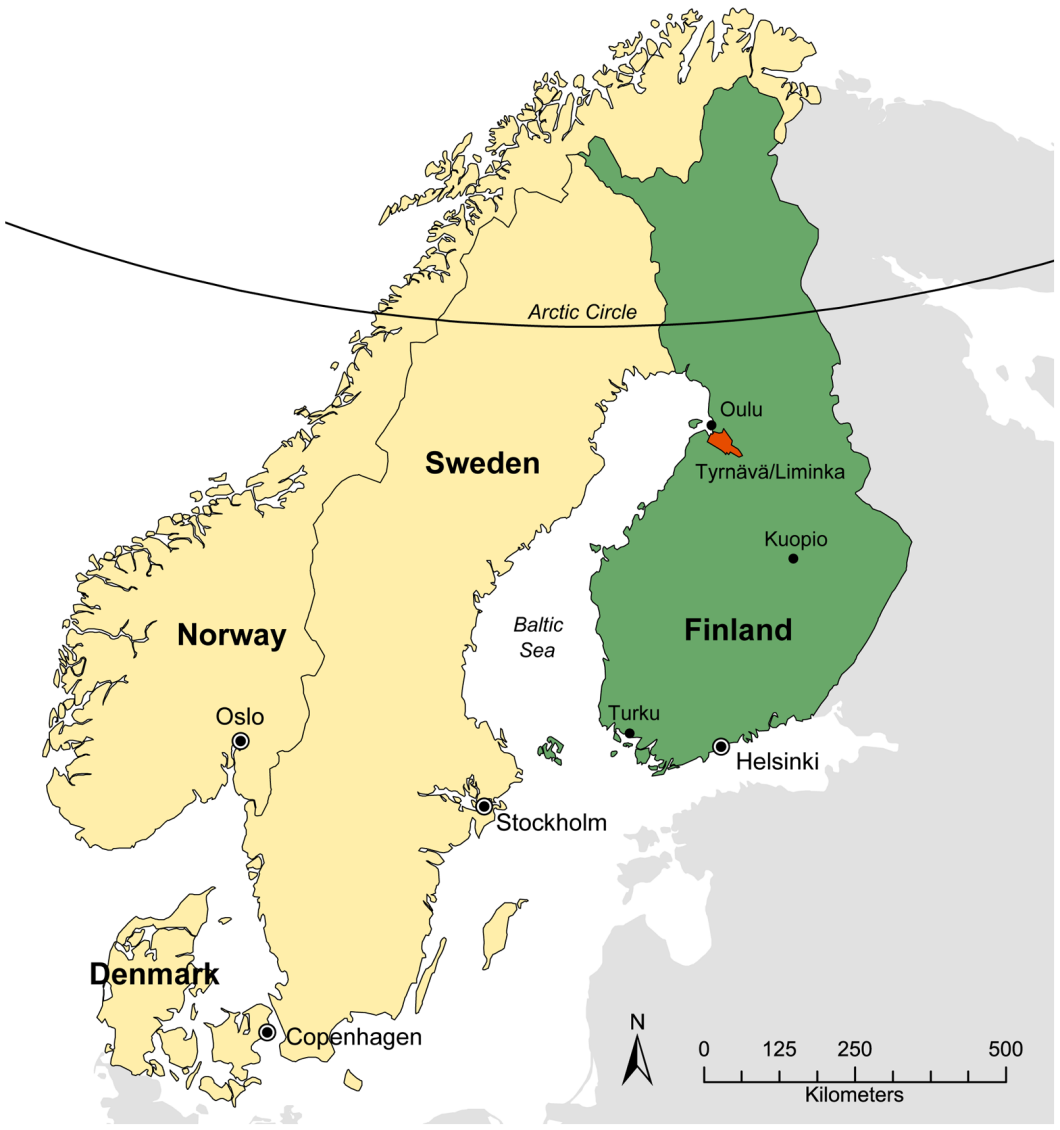
Map of the Nordic countries. The region under study (Tyrnävä-Liminka area; 64°46N, 25°32E; ca 100 km^2^) in Finland is shown in red.

**Figure 2:**
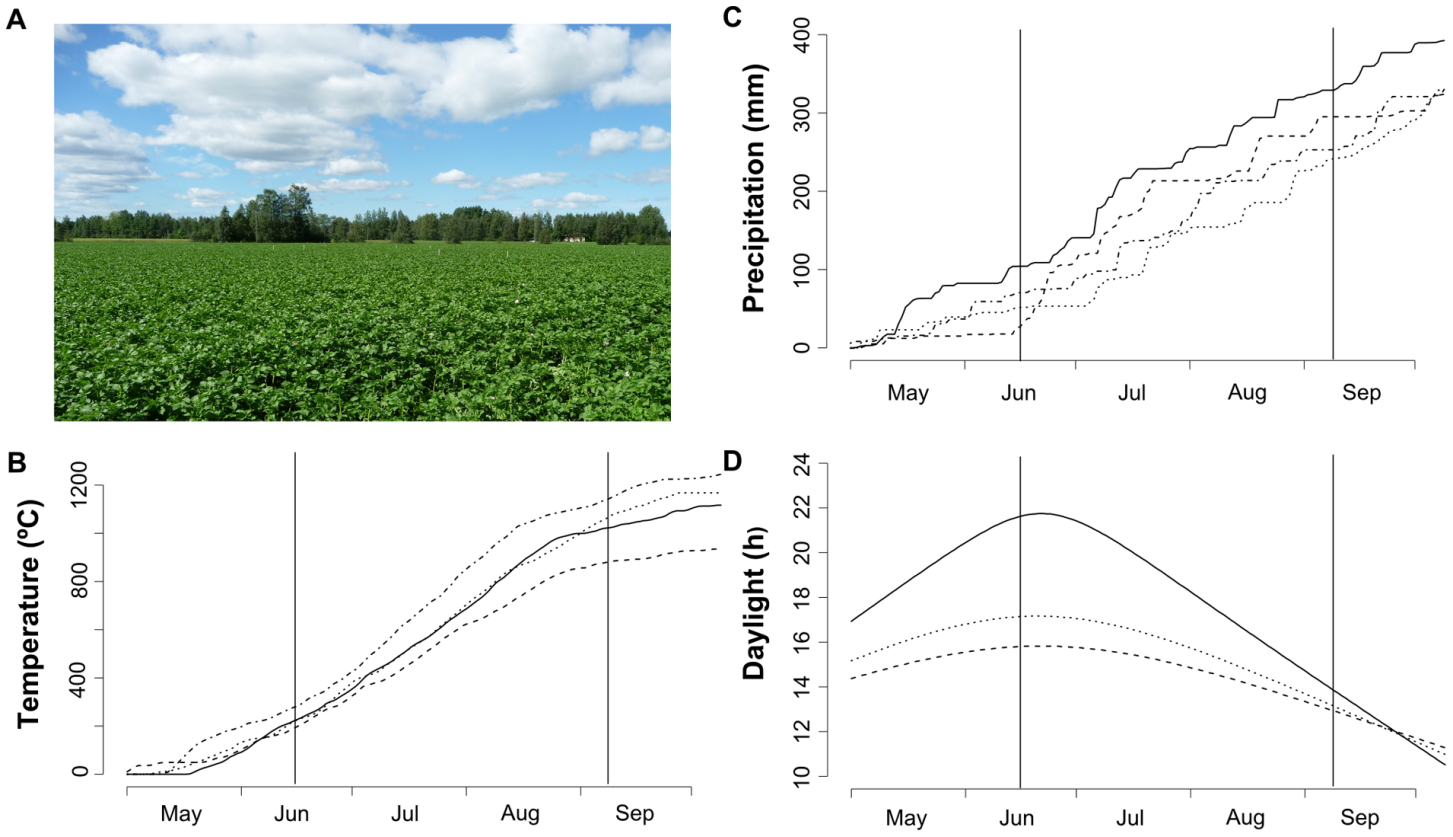
Climatic conditions in the Tyrnävä-Liminka area in Finland during the thermal growing seasons in 2007–2010. (A) A view on a typical agricultural landscape and seed potato crop. (B) Cumulative effective temperature (*T*_base_  = 5°C) and (C) cumulative precipitation over the thermal growing seasons. The years are depicted by solid (2007), dashed (2008), dotted (2009) or dotdashed (2010) lines. (D) Comparison of hours of photosynthetically active daylight in three important seed potato production areas: Tyrnävä-Liminka, Finland (solid line), Rostock, Germany (dotted line), and New Brunswick, Canada (dashed line). Vertical lines in (B) to (D) define the potato growing season in Tyrnävä-Liminka area.

The study was carried out in the Tyrnävä-Liminka area (64°46N, 25°32E; ca 100 km^2^) in the coastal zone of the Gulf of Bothnia, 2–10 m above sea level (Fig. 1) in 2007–2010. The mean thermal growing season (*T*_base_  = 5°C) begins on May 1 and ends on October 9 (average of years 1981–2010) [18]. A typical view on the agricultural landscape, respective cumulative effective temperature, cumulative precipitation and daylight hours are shown in Figure 2. The landscape was structured by agricultural areas surrounded by young to middle-aged mixed forest (*Betula* spp., *Pinus* spp. and *Picea* spp.).

### Aphid monitoring

In four years (2007–2010), aphid flights were monitored over the potato growing season, which starts in mid-June and ends in the beginning of September in the Tyrnävä-Liminka area. Yellow pan traps (YPT) (Syngenta Agro GmbH, Maintal, Germany) were used to study local occurrence of aphids in 4 to 8 fields per year. YPTs were 27.0×33.0×8.0 cm (width x length x height) and filled with ca. 1.5 l of tap water containing 1 ml of 50% Tween20 as an odourless detergent. Single YPTs per field were used in 2007, whereas in 2008–2010, three YPTs were used in each field. The three YPTs were arranged in a triangle (distance 1.5 m between YPTs) [9] and the total catch of aphids from them was used for analysis because of the low aphid abundance. The number of aphids caught in 2007 was multiplied by a factor of 2.79 to make data comparable to years 2008–2010. The factor was determined by a calibration experiment in 2008 (see [9] for further details). Each trap was placed on a black box (60×40×16 cm) turned upside down and situated at an edge of a potato field in the middle of an area of 4×4 m maintained as a bare fallow by herbicide treatments to ensure uniformity of the conditions for trapping over sites and periods of time [19]. YPTs were emptied twice a week. All YPTs were replaced by new ones in the middle of the potato growing season, because bleaching of the yellow colour of traps may occur gradually upon exposure to sunlight [20] and influence the landing response of aphids [21],[22].

The flight activity of aphids was monitored using a 12.2 m high Rothamsted suction trap [23] centrally located in the research area. The suction trap was emptied daily.

### Species identification

Aphid catches from both types of trap were stored in 70% ethanol. Aphids were identified using a microscope (50–100X magnification) and various taxonomic keys [24],[25],[26],[27],[28],[29],[30]. Individuals which could not be identified to species were grouped at genus level. *Aphis fabae* s.s. and *Aphis fabae* subspecies were not separated [26] but assigned to *Aphis fabae* group. Grouping of species according to the life cycle was done based on a host plant catalogue of aphids [31].

DNA barcoding was done to complement morphological identification. Total DNA was extracted from single aphid individuals using DNeasy Blood & Tissue Kit (Qiagen, Hilden, Germany) according to the manufacturer's supplementary protocol for insects. Barcoding was done based on a 658-bp long region of the mitochondrial gene for cytochrome *c* oxidase I (*COI*) amplified using primers LepF (5′-ATTCAACCAATCATAAAGATATTGG-3′) and LepR (5′-TAAACTTCTGGATGTCCAAAAAATCA-3′) [32]. PCR was done in a total volume of 50 μl and contained 5% trehalose, 10 mM Tris-HCl (pH 8.8), 50 mM KCl, 0.1% Triton X-100, 3 mM MgCl_2_, 5 pmol of each primer, 1.4 units Dynazyme II (Finnzymes, Espoo, Finland), 50 µM dNTP, and 10–50 ng DNA. The PCR programme consisted of a 2 min pre-incubation at 94°C, followed by 40 cycles of 40 seconds at 94°C, 40 seconds at 49°C and 1 min at 72°C. Final extension was done for 5 min at 72°C.

PCR products were detected on 1.0% Tris-acetate-EDTA agarose gels by electrophoresis followed by staining with ethidium bromide. PCR products of the expected size were sequenced in both directions with LepF and LepR primers by the Sequencing Laboratory of the Haartman Institute, University of Helsinki, Finland. BioEdit software was used for evaluating the quality of sequence data and also for the alignment of sequences. Sequences and additional information on voucher material were uploaded to the Barcode of Life Data (BOLD) Systems database (http://www.boldsystems.org; project code ‘AFNF’). Vouchers are available at the Finnish Museum of Natural History/Zoology Unit, Helsinki, Finland, under accession numbers AFNF001-12 to AFNF0039-12.

Sequences obtained in this study were compared with sequences obtained from the BOLD Systems database. For sequence alignment, longer sequences were cut to the standardised length of 658 bp of the barcode region of *COI*. Sequences which were shorter than 658 bp or had missing values were excluded from the alignment. Neighbour-joining analysis of sequences was done with Mega4 software, as described [33].

### Analysis of data

Characterisation of seasonal flight patterns was based on the catch from YPTs. Aphid species represented by less than 10 individuals in the total catch of the year were omitted. Data preparation was done in three steps to ensure that all traps contributed equally to an averaged seasonal flight pattern of a species and that traps with high numbers of aphids did not overly dominate phenology characteristics of traps with low overall aphid numbers [34],[35]. First, for each week (*k*) and species (*e*) for a particular trap [site-by-year combination (*i*)], counts of individuals of a species (*c*) were divided by the total count of the species for the site-by-year combination (eqn1) concerned, resulting in a standardised count (*t_kei_*).
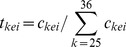
(1)


Second, resulting seasonal flight patterns were averaged over all traps (*i*) (site-by-year combinations) per species and denoted as (*n_ke_*)_,_ (eqn 2),
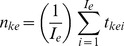
(2)where (*I_e_*) is the total number of site-by-year combinations for which there was at least one individual in species (*e*). In other words, the (*n_ke_*) average includes only those site-by-year combinations (*i*) for which species (*e*) was present. Third, the resulting averages (*n_ke_*) were normalised to their respective peaks to allow comparisons between species.



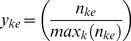
(3)Normalised and averaged flight patterns (*y_e_*) were then analysed by hierarchical clustering generated with the R package ‘Pvclust’ [36] in R, version 2.15.1 [37]. The procedure was performed with multiscale bootstrap with 10,000 repetitions, average linkage for cluster joining and a maximum-based dissimilarity matrix. Approximately Unbiased (AU) probability values were computed using bootstrap samples of various sizes to test how strongly the cluster was supported (AU>95%). Flight patterns of species that clustered together were averaged over species (eqn 4) and normalised to their respective peak (eqn 5) to obtain typical phenology patterns,
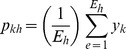
(4)where (*h*) is the cluster and (*E*) the number of species in that cluster.



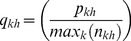
(5)


## Results

### Aphid fauna

A total of 58,528 winged aphid individuals were caught in 2007–2010, including 56,040 aphids obtained with YPTs and 2488 individuals with the suction trap. The number of taxa was 83, of which 54 taxa could be identified to the species level and the remaining 29 taxa at a genus level. A small proportion of aphids (0.3–3.3% per year) could not be identified (Table 1). Seventy-four taxa were recorded in the YPTs, including 23 taxa exclusively in them. On the other hand, the suction trap caught 61 aphid taxa, of which ten taxa were not detected in the YPTs. Barcoding of *COI* was done for 34 aphid species (Table S1). *Calaphis betulicola, Cryptomyzus stachydis* and *Myzus padellus* were barcoded for the first time.

**Table 1:** Relative abundance of aphid taxa in the suction trap (ST) and yellow pan traps (YPT) in Tyrnävä-Liminka area, Northern Finland, in 2007–2010.

			2007		2008		2009		2010	
	Life cycle	Cluster	ST	YPT	ST	YPT	ST	YPT	ST	YPT
Total individuals			60	2925	364	6257	961	30013	1103	16845
*Acyrthosiphon pisum* (Harris, 1776)*	m1	B1	1.67	0.67	0.55	0.85	0.10	0.31	0.54	0.60
*Acyrthosiphon* spp.	m		-	-	0.27	-	-	0.01	-	0.01
*Adelges* spp.			-	-	-	-	0.21	-	**-**	-
*Amphorophora rubi* (Kaltenbach, 1843)*	m2	A1	-	0.29	-	1.57	-	0.03	**-**	-
*Anoecia* spp.			-	-	-	0.13	0.10	-	**-**	-
*Aphis fabae* group*	h	A2	3.33	9.35	1.10	10.61	0.83	8.14	0.27	0.94
*Aphis frangulae*/*gossypii* complex^1^*	h	A1	-	0.67	0.55	1.69	-	0.87	**-**	0.23
*Aphis idaei* van der Goot, 1912*	m2	A1	-	2.48	-	2.00	-	1.32	**-**	0.36
*Aphis pomi* deGeer, 1773*	m2	A1	1.67	0.76	-	0.66	-	0.08	**-**	0.02
*Aphis salicariae* Koch, 1855	h		-	-	1.10	-	1.46	-	**-**	-
*Aphis sambuci* Linnaeus, 1758	h	A1	-	0.38	-	0.26	-	0.18	**-**	0.02
*Aphis* spp.	hm	A1	-	1.81	0.27	2.05	1.25	1.10	**-**	0.63
*Aulacorthum solani* (Kaltenbach, 1843)*	h	A1	-	0.19	2.75	11.28	0.31	0.36	0.09	0.12
*Brachycaudus* spp.	hm	B1	-	-	0.27	0.08	0.21	0.03	0.27	0.02
*Calaphis betulicola* (Kaltenbach, 1843)*	m2	A2	-	1.34	0.55	0.62	0.42	1.29	0.09	0.13
*Calaphis flava* Mordvilko, 1928*	m2	A1	-	11.16	-	0.99	2.91	4.80	0.09	0.19
*Callipterinella minutissima* (Stroyan, 1953)	m2		-	-	0.27	-	-	0.01	-	-
*Capitophorus hippophaes* (Walker, 1852)*	h	B2	-	0.19	-	2.00	0.10	0.44	-	0.06
*Capitophorus similis* van der Goot, 1915	h	A1	-	-	-	1.15	0.21	1.54	-	-
*Capitophorus* spp.	hm		-	-	0.27	0.08	-	-	-	-
*Cavariella aegopodii* (Scopoli, 1763)*	h	A1	1.67	8.21	0.27	1.15	0.10	0.22	-	0.04
*Cavariella archangelicae* (Scopoli, 1763)	h	A1	-	-	0.00	0.03	0.10	0.22	-	0.01
*Cavariella intermedia* Hille Ris Lambers, 1969			-	-	-	0.03	-	-	-	-
*Cavariella konoi* Takahashi, 1939*	h	A1	-	-	-	0.13	0.52	0.42	0.09	-
*Cavariella pastinacae* (Linnaeus, 1758)*	h	A1	3.33	0.95	0.55	0.89	0.52	4.33	-	0.04
*Cavariella theobaldi* (Gillette & Bragg, 1918)*	h	A1	-	0.10	0.27	0.26	-	0.66	-	-
*Chaitophorus* spp.	m2	A1	1.67	0.10	-	1.98	2.81	13.85	0.18	0.02
*Cinara* spp.	m2		-	-	-	0.03	0.42	0.00	0.18	0.01
*Cryptomyzus galeopsidis* (Kaltenbach, 1843)*	h	B2	-	13.45	7.14	11.30	2.29	7.24	0.18	3.14
*Cryptomyzus korschelti* Börner, 1938	h	B2	-	0.76	-	0.43	0.10	0.10	-	-
*Cryptomyzus ribis* (Linnaeus, 1758)	h	B2	-	0.76	-	0.16	-	0.05	-	0.06
*Cryptomyzus stachydis* (Heikinheimo, 1955)*	h	A1	-	0.10	-	0.46	-	0.02	-	-
*Diuraphis* spp.	m1	B1	-	1.15	0.27	0.14	0.10	0.14	-	0.13
*Dysaphis* spp.	h^2^	B2	-	-	-	1.50	0.10	0.19	-	-
*Elatobium abietinum* (Walker, 1849)*	m2		-	-	-	-	1.77	-	-	-
*Euceraphis* spp.	m2	A1	5.00	0.48	3.02	0.05	2.50	0.28	90.12	1.55
*Eulachnus* spp.	m2		-	-	-	-	0.10	-	-	-
*Hayhurstia atriplicis* (Linnaeus, 1761)*	m1	B1	-	26.15	-	3.64	0.42	6.87	1.54	87.09
*Hyalopterus pruni* (Geoffroy, 1762)*	h	B2	-	0.10	9.62	0.45	1.25	0.00	0.36	0.10
*Hyperomyzus lactucae* (Linnaeus, 1758)*	h	B1	1.67	5.15	1.10	3.36	0.31	1.39	-	0.21
*Hyperomyzus pallidus* Hille Ris Lambers, 1935	h	B2	-	0.19	-	0.21	-	0.02	-	-

	Life cycle	Cluster	ST	YPT	ST	YPT	ST	YPT	ST	YPT
Total individuals			60	2925	364	6257	961	30013	1103	16845
*Hyperomyzus rhinanthi* (Schouteden, 1903)*	h	B2	1.67	0.10	0.55	0.38	1.04	1.20	0.09	0.04
*Hyperomyzus* spp.	hm		-	-	-	0.02	-	-	-	-
*Illinoia* spp.	m^3^		-	0.10	-	-	-	-	-	-
*Macrosiphoniella* spp.	m1	B1	6.67	0.19	-	0.18	0.10	0.03	0.09	0.05
*Macrosiphum euphorbiae* (Thomas, 1878)*	h	B1	1.67	1.91	-	0.56	0.21	0.29	-	0.11
*Macrosiphum gei* (Koch, 1855)	m1		-	-	-	0.03	-	-	-	-
*Macrosiphum rosae* (Linnaeus, 1758)*	h	A2	-	-	1.10	0.05	0.21	0.04	-	0.01
*Macrosiphum* spp.	m^3^		-	-	-	0.02	-	0.00	-	-
*Megoura viciae* Buckton, 1876*	m1	B1	-	-	-	0.02	0.10	0.06	0.09	0.11
*Metopolophium dirhodum* (Walker, 1849)*	h	B1	16.67	3.44	3.30	7.64	1.35	0.88	0.18	0.14
*Metopolophium festucae* (Theobald, 1917)	m1		-	0.10	-	-	-	-	-	-
*Microlophium carnosum* (Buckton, 1876)	m1	B1	-	-	-	-	-	-	-	0.06
*Myzaphis rosarum* (Kaltenbach, 1843)*	m2	A1	-	-	-	0.02	-	0.08	-	-
*Myzodium modestum* (Hottes, 1926)	m^4^		-	-	-	-	0.21	-	-	-
*Myzus padellus* H.R.Lambers & Rogerson,1946*	h	A1	-	-	2.75	1.61	21.12	7.54	0.45	0.31
*Myzus persicae* (Sulzer, 1776)*	h	B1	-	-	-	0.08	-	0.06	-	0.09
*Myzus* spp.	hm	B2	-	0.29	0.82	0.26	0.10	0.03	-	0.01
*Nasonovia pilosellae* (Börner, 1933)	m1		-	-	-	-	-	0.01	-	-
*Nasonovia ribisnigri* (Mosley, 1841)*	h	B1	-	0.19	-	0.05	-	0.04	-	0.02
*Pemphigus* spp.	h^2^		-	-	-	0.06	0.52	-	-	-
*Periphyllus aceris* (Linnaeus, 1761)	m2		-	-	-	0.02	-	-	-	-
*Phorodon humuli* (Schrank, 1801)*	h		-	-	-	-	0.10	-	-	-
*Prociphilus* spp.	h		-	-	-	-	0.52	-	-	-
*Protrama* spp.	m1	B1	-	0.86	-	0.30	-	0.30	-	0.15
*Pterocomma* spp.	m2	A1	-	-	-	0.18	-	0.20	-	0.02
*Rhopalomyzus lonicerae* (Siebold, 1839)*	h		-	-	0.27	-	-	-	-	-
*Rhopalosiphoninus* spp.			-	-	-	0.10	-	-	-	0.01
*Rhopalosiphoninus staphyleae* (Koch, 1854)	h		-	-	0.27	-	-	0.00	-	-
*Rhopalosiphum padi* (Linnaeus, 1758)*	h	B2	33.33	1.62	47.80	22.89	43.91	29.45	3.63	0.48
*Schizaphis* spp.	m1		-	0.10	0.27	-	0.21	0.01	-	0.01
*Semiaphis* spp.	hm		-	-	-	-	-	0.01	-	-
*Sitobion avenae* (Fabricius, 1775)*	m1	B2	6.67	0.10	5.77	0.02	4.37	0.04	0.54	0.04
*Sitobion fragariae* (Walker, 1848)	h		1.67	0.19	0.27	-	0.21	-	-	-
*Sitobion* spp.	hm		-	-	0.27	-	-	-	-	-
*Tetraneura ulmi* (Linnaeus, 1758)	h	B2	1.67	0.10	0.55	0.03	0.21	0.00	-	0.01
*Thecabius affinis* (Kaltenbach, 1843)	h	A1	-	-	1.92	0.03	0.73	1.20	0.27	0.09
*Therioaphis* spp.	m1		-	-	-	-	0.21	0.00	-	0.11
*Therioaphis trifolii* (Monell, 1882)	m1		-	-	-	0.02	-	-	-	-
*Thripsaphis cyperi* (Walker, 1848)	m1	A1	1.67	-	0.55	-	1.77	0.06	-	0.02
*Thripsaphis* spp.	m1		-	-	0.55	0.02	-	0.01	-	-
*Uroleucon* spp.	m1	B1	-	0.86	-	2.21	0.42	1.54	0.09	2.08
*Wahlgreniella vaccinii* (Theobald, 1924)	m2		5.00	-	-	-	-	-	-	-
other aphids			3.33	2.96	2.75	1.02	0.83	0.36	0.54	0.33

***m***: non host-alternating; possible species comprise both *m1* and *m2*. ***m1***: non host-alternating on herbaceous host. ***m2***: non host-alternating on woody host. ***h***: host-alternating. ***hm***: no indentification to species level. **ST**: suction trap. **YPT**: Yellow pan trap. **^1^** morphological indistinguishable; barcoded individuals were *Aphis gossypii* Glover, 1877. **^2^**species in this genus are mostly host-alternating. **^3^**species in this genus mostly non host-alternating. **^4^**non host-alternating on mosses. *Species further characterised by DNA barcoding; *COI* sequences are available in the Barcode of Life Data (BOLD) systems database (http://www.boldsystems.org); accession numbers AFNF001-12 to AFNF0039-12.

In addition, six *Aphis gossypii* individuals obtained from different fields in 2008-2010 were barcoded and the *COI* sequences found to be identical. They were and also nearly identical to the 101 *Aphis gossypii COI* sequences available in the database. They were clearly different from other members of the tribe Aphidini, such as *Aphis frangulae* and *Aphis fabae* included for comparison (Fig. S1). Furthermore, DNA was isolated, pooled and the *COI* barcode sequence determined for an additional 30 aphid individuals, which were caught from different fields in different years and identified to *Aphis gossypii* based on morphological characteristics. The sequence obtained was identical to the sequences of the six individually bar-coded aphids of *Aphis gossypii* and no double peaks were observed in the phenogram, indicating that most if not all pooled individuals had an identical *COI* sequence. Taken together, these data indicated that *Aphis gossypii* occurred frequently in the field (Table 1).

**Figure 3:**
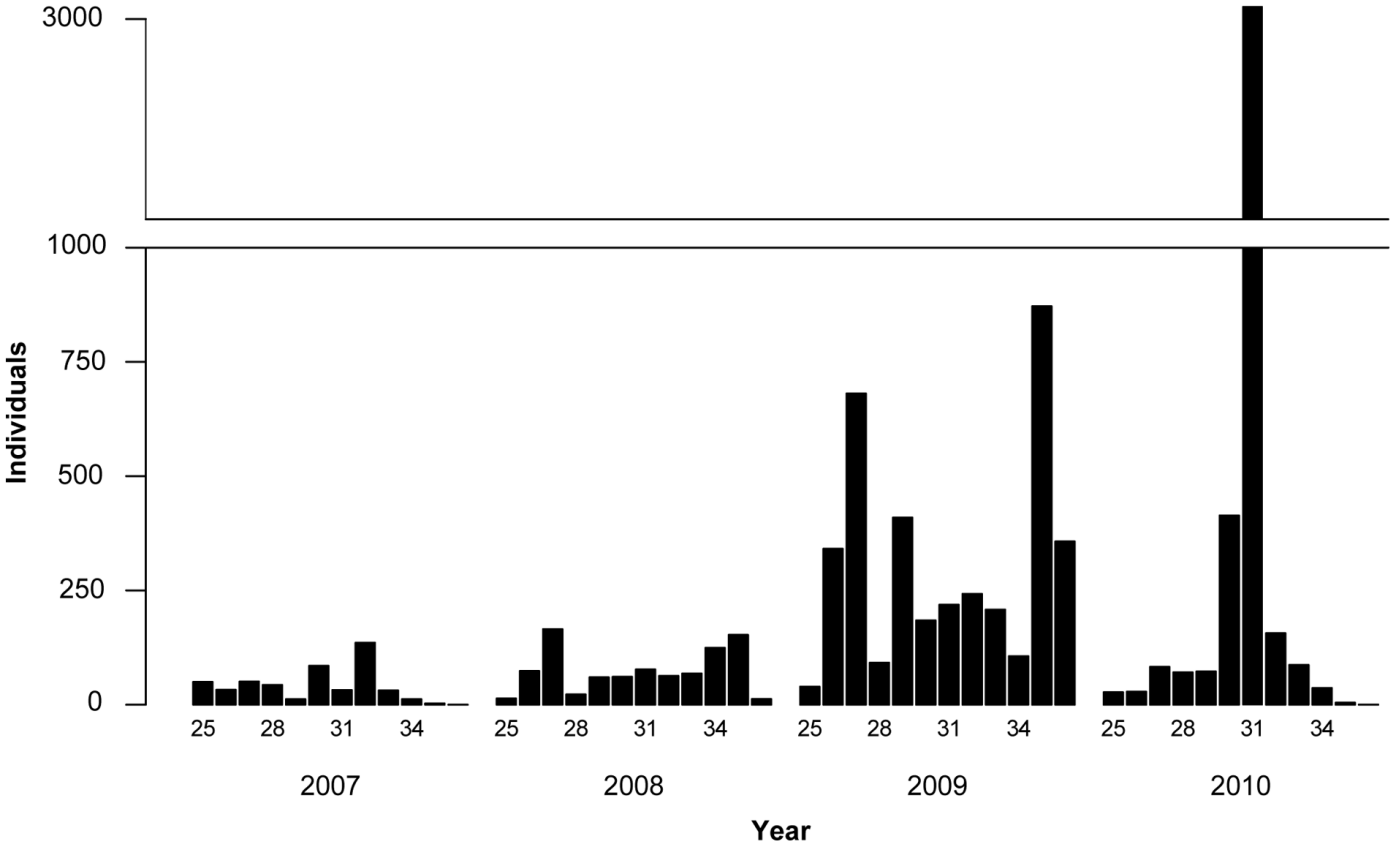
Weekly numbers of winged aphids caught with yellow pan traps (YPT). Aphids were monitored with YPTs over the potato growing season from mid-June to the beginning of September (weeks 25–36) in Tyrnävä-Liminka area, Finland, in 2007–2010. Each bar indicates the total number of aphids obtained in the given week from all fields monitored with YPTs. The numbers of fields were 6 (2007), 7 (2008), 8 (2009) or 4 (2010). The number of every third calendar week is shown below bars.

**Figure 4:**
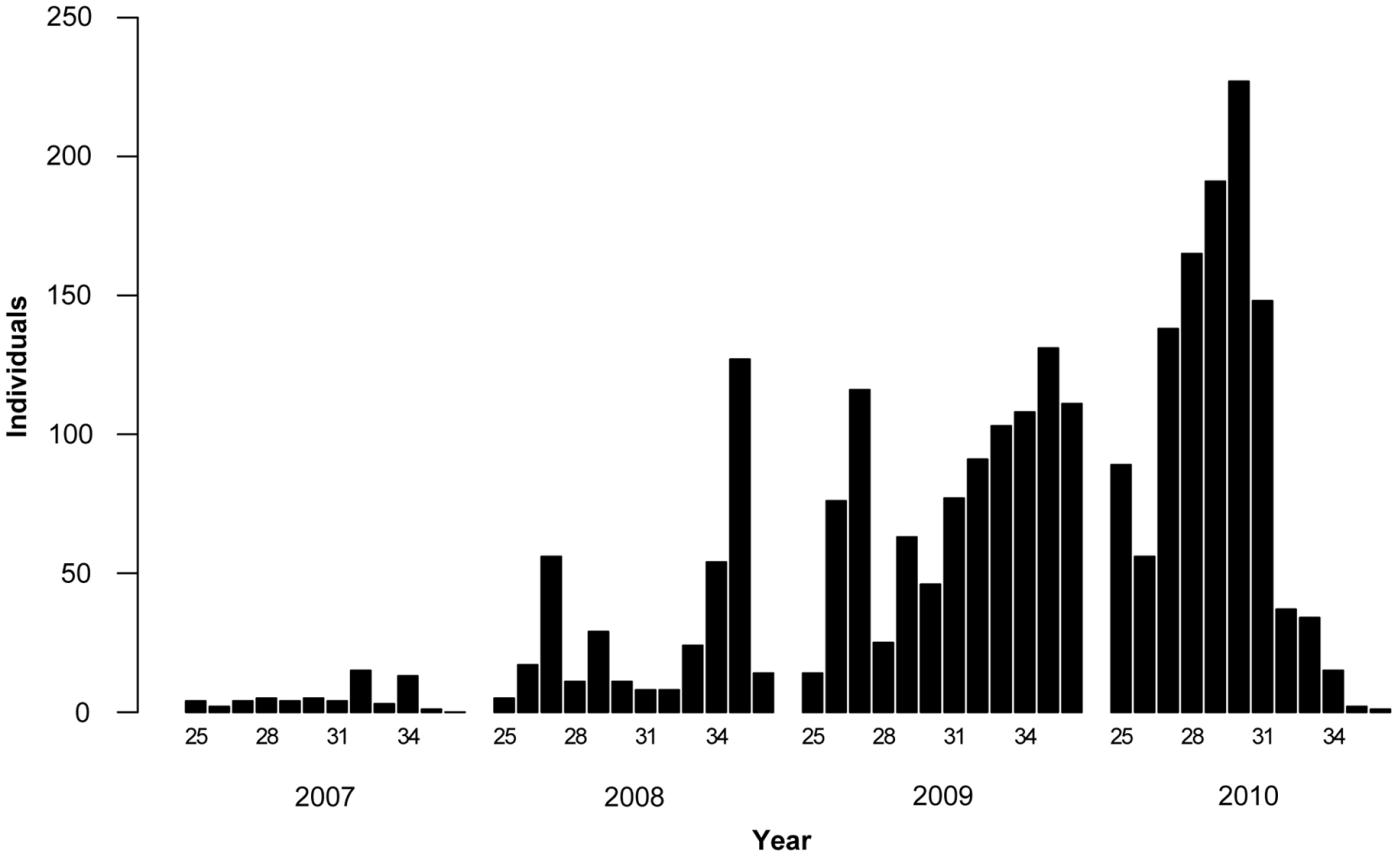
Weekly numbers of winged aphids caught with a suction trap. Aphids were monitored with the suction trap over the potato growing season from mid-June to the beginning of September (weeks 25–36) in Tyrnävä-Liminka area, Finland, in 2007–2010. Each bar indicates the total number of aphids obtained in the given week. The number of every third calendar week is shown below bars.

The spectrum of aphid species in the four years was dominated by the family Aphididae, the subfamily Aphidinae and the tribe Macrosiphini (79%, 75% and 63% of the total number of taxa, respectively) (Table 1). Monitoring of aphids with YPTs in the field and using the suction trap provided consistent data in terms of the general abundance of aphids in the region (Figs. 3, 4). Furthermore, the most abundant species was usually the same in both trap types. For example, *Rhopalosiphum padi* dominated in YPT catches in 2008 and 2009 (22.9% and 29.5%, respectively) and in the suction trap in 2007, 2008, and 2009 (33.3%, 47.8% and 43.9%, respectively). However, *Hayhurstia atriplicis* was the most prevalent species caught with YPTs in 2007 and 2010 (26.2% and 87.1%, respectively), but was not observed in such proportionally high numbers in the suction trap. Other agriculturally important pest species in the YPT catch were *Aphis fabae* (6.3% of the total catch in 2007–2010), *Aphis gossypii* (0.8%), *Macrosiphum euphorbiae* (0.3%) and *Myzus persicae* (0.1%).

**Figure 5:**
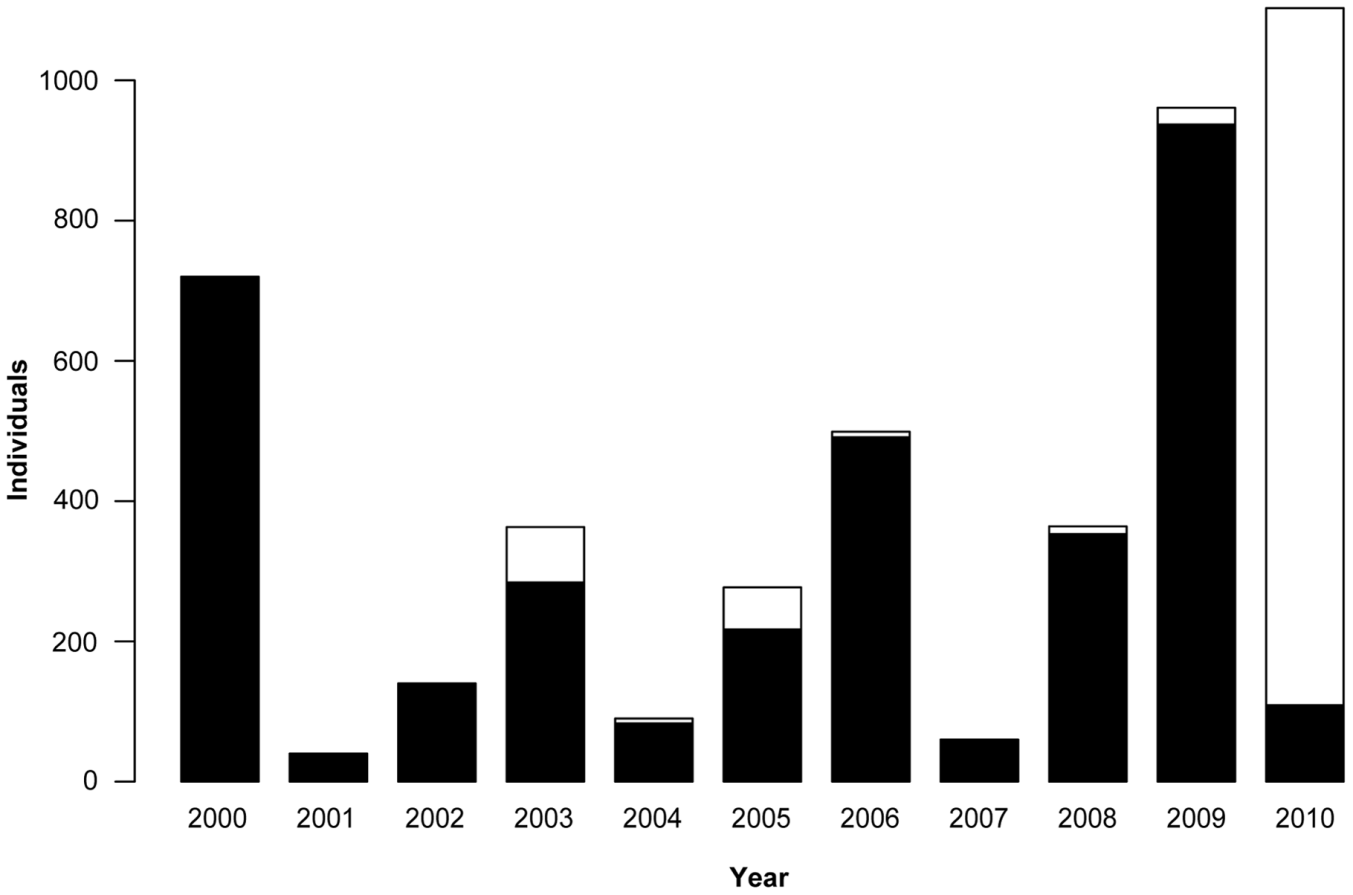
Total annual number of winged aphids caught with a suction trap in Tyrnävä-Liminka area, Northern Finland, in 2000–2010. Monitoring was done during potato growing seasons from mid-June to beginning of September. White segments in stacked bars depict proportions of *Euceraphis* spp. For the years 2000–2002 only data on total numbers of aphids were available.

Analysis of the numbers of aphids obtained with the suction trap over 11 years, including the period of this study and the data available from seven previous years, revealed a recurring three-year cyclic pattern that was apparent when the exceptionally high numbers of *Euceraphis* spp. caught with the suction trap in 2010 were excluded from data (Fig. 5).

### Phenological characteristics

**Figure 6:**
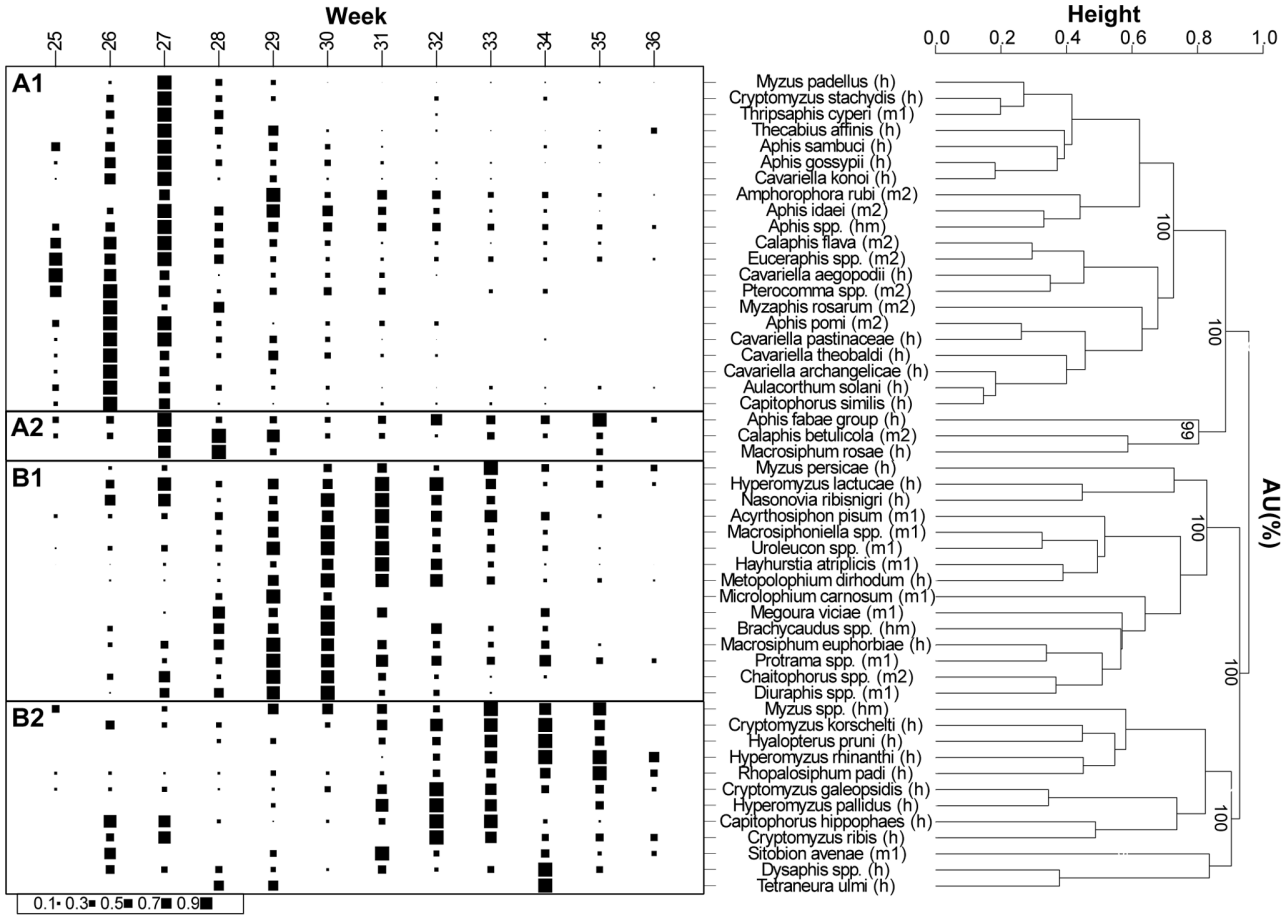
Clustering analysis of aphid flight patterns. Clustering was based on normalised abundance of 51 aphid taxa monitored with yellow pan traps in a total of 25 potato fields during four growing seasons (2007–2010). Relative abundances of aphid species in a given calendar week (column) is indicated by the sizes of black squares. Patterns were grouped using hierarchical clustering ‘pvclust’ package in R. Approximately unbiased (AU) probability values (%) are shown in the cluster dendrogram to the right. Clusters with AU>95% are strongly supported by data. Life cyle codes shown in parentheses at the end of the species name: *h*  =  heteroecious species; *m1*  =  monocious species on herbaceous plants; *m2*  =  monocious species on trees and shrubs; *hm*  =  not indentified at species level – potential species in this genus could be host-alternating or non host-alternating.

**Figure 7:**
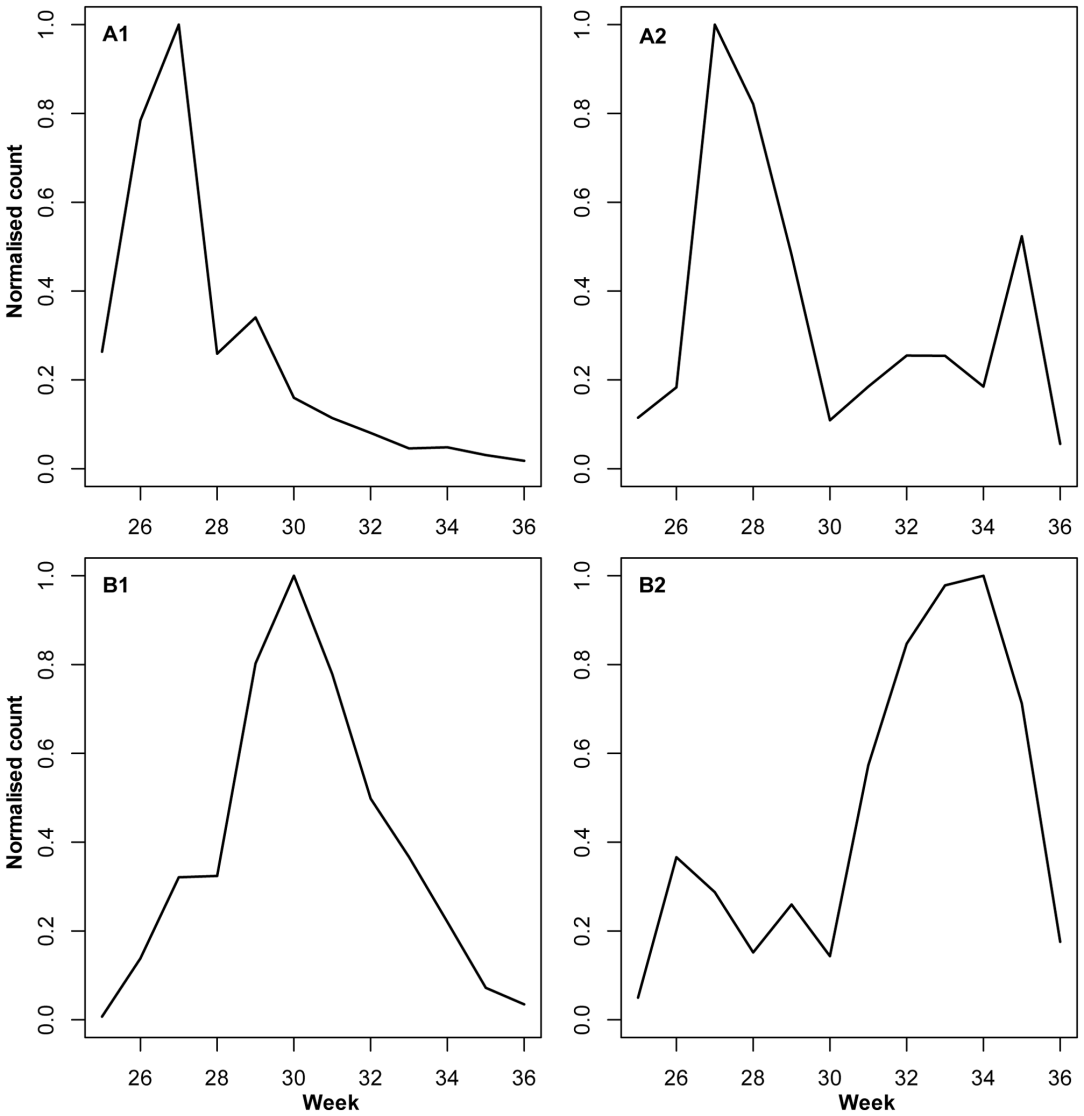
Four phenology clusters characterised by different seasonal timing of aphid flight activity. Clusters were determined by hierarchical cluster analysis based on normalised abundance of 51 aphid taxa monitored with yellow pan traps in a total of 25 potato fields during four growing seasons (2007–2010). The numbers of taxa belonging to the phenology clusters: A1, 21 taxa; A2,: 3 taxa; B1, 15 taxa; and B2, 12 taxa.

A cluster dendrogram (Fig. 6) based on YPT data and normalised abundance of single species during the potato growing season revealed that the 51 most abundant taxa used for the analysis fell into three main phenology clusters corresponding to early (cluster A1), mid-term (B1) and late (B2) flight activity. An additional smaller cluster (A2) contained species showing flight activity early and late during the potato growth season (Fig. 7). All clusters showed high Approximately Unbiased (AU) scores (>95%).

Among these 51 analysed taxa, 29 taxa were heteroecious (host-alternating) moving from trees/shrubs to herbaceous plants. Ten taxa were monoecious (non host-alternating) living on herbaceous plants, and nine taxa were monoecious living on trees/shrubs. Three taxa were identified at genus level only and cannot be assigned to any of these life cycles, because the genera include monoecious and heteroecious species. The early and late flight patterns were only seven weeks apart, with maximum flight activities being in weeks 27 and 34, respectively (Figs. 6,7). The early flight clusters A1 and A2 comprised eight monoecious taxa living on trees/shrubs and 14 heteroecious taxa. The mid-term flight cluster B1 comprised eight monoecious taxa, which use herbaceous plant species as hosts, and five heteroecious taxa. Late flight activity (B2) was characteristic of nine heteroecious taxa (Fig. 6).

*Aphis* spp. and *Cavariella* spp. were found in cluster A1, except *Aphis fabae* that was located to cluster A2 (Fig. 6). *Hyperomyzus* species were found in clusters B1 and B2. *Cryptomyzus* was distributed to clusters A1, B1 and B2 depending on species. The important pests *Acyrthosiphum pisum, Macrosiphum euphorbiae*, *Metopolophium dirhodum* and *Myzus persicae* were placed to cluster B1, whereas *Sitobion avenae* and *Rhopalosiphum padi* occurred late and were placed to cluster B2 (Fig. 6).

## Discussion

In this study, seasonal flight activity of aphids was characterised in the northernmost High Grade seed potato growing zone in Europe, in a region with intensive agricultural production. The majority (80%) of the non host-alternating aphid taxa on herbaceous hosts were found to have a pronounced peak in their occurrence in the middle of the potato growing season, whereas 90% of the non host-alternating taxa on woody hosts showed a peak of occurrence early in the potato growing season. Late-season flight activity was characteristic of the host-alternating species.

Among the agriculturally most relevant aphid species [38] found in the area, the cereal aphids *Sitobion avenae* and *Rhopalosiphum padi* occured late in the growing season. *Rhopalosiphum padi* showed a distinctive monomodal flight activity in contrast to the bimodal pattern reported in France [35] or multimodal pattern found in North-central parts of the U.S.A. [39]. These aphid species may be of minor relevance as virus vectors in Tyrnävä-Liminka area, in line of our previous study [9], because development of mature plant resistance makes potato plants less susceptible to virus infections towards the end of the growing season [6],[40]. The abundance of *Rhopalosiphum padi* in the northern parts of Finland is generally lower and the flight activity occurs later than in southern Finland [14],[41] where this species is considered as one of the main vectors of the barley yellow dwarf virus complex [42] causing important diseases in cereals [43].

*Aphis fabae* contributed considerably (6.3%) to the total YPT catch. This species was characterised by two major flight periods at the beginning and the end of the potato growing season. Potato plants are most susceptible to virus infection at early growth stages prior to flowering [6],[40]. The early flight activity of *Aphis fabae* during a young growth stage of field crops in Tyrnävä-Liminka area, the polyphagous nature of the species, and its ability to vector more than 30 plant viruses, including many non-persistently transmitted viruses [44],[45], renders *Aphis fabae* as a potential key pest species in the northern agricultural region of this study. Indeed, *Aphis fabae* appears to be the main vector of *Potato virus Y* in potato crops in the region [9]. The snowball tree (*Viburnum opulus* L.) is the only known winter host of *Aphis fabae* in the Tyrnävä-Liminka area [9].

Previous studies have considered *Aphis gossypii* as a subspecies of the *Aphis frangulae* complex in Europe [26],[46], but recent molecular studies indicate that it represents a genetically distinguishable species [47],[48]. Studies on samples from a broad geographical area indicate that intraspecific genetic variability of *Aphis gossypii* is low [33]. No differences were observed in the *COI* barcode sequences among individuals of *Aphis gossypii* characterised in our study, but they differed from all individuals of *Aphis gossypii* characterised from other countries for at least one nucleotide substitution in *COI*. There is currently no information about potential primary hosts of *Aphis gossypii* in Europe. Therefore, European populations are considered to be anholocyclic and to overwinter parthenogenetically in protected places [38]. This study found that in all years *Aphis gossypii* occured in noticeable numbers (0.8% of the total catch) in the field, in contrast to previous reports suggesting that it occurs exclusively indoors and should be considered as a glasshouse pest only [26],[49]. Our results suggest that greenhouse populations are able to establish well on wild secondary hosts, even far in north in Europe.

In a recent risk assessment study about alien species in Finland, *Aphis gossypii* was ranked in the highest category of potential future pests [50]. It is known to transmit 75 plant viruses [51] and colonise more than 600 host plants [52]. Our study shows that the current pest status of this extremely polyphagous species needs to be re-evaluated. To do this, further research is needed in order to establish whether factors such as long distance migration or unknown primary host plant relations are involved in the population dynamics of this species. In addition, in light of the warming climate, it will be essential to assess the response of this species to climatic conditions in the field.

In 2010, an exceptionally high proportion (90%) of the catch in the suction trap consisted of *Euceraphis* spp. (Fig. 5; Table 1). A similar outbreak of *Euceraphis* spp. in Finland occurred in 1988 [53]. Those exceptional abundances were attributed to long distance migration caused by strong eastern wind gusts from birch forests in Russia and Belarus [53]. However, in YPTs *Euceraphis* spp. were observed only in small numbers (1.5% of the total catch). In contrast, *Hayhurstia atriplicis* occurred in large numbers in YPTs but only few individuals of this species were caught with the suction trap. These differences may be associated with the height at which the species are normally flying. *Euceraphis* spp. is a tree-dwelling species and may prefer to fly higher than *Hayhurstia atriplicis* whose secondary hosts are species of *Chenopodiaceae* and whose catch is known to be significantly affected by trap height [54]. Another influencing factor could be that suction traps measure the absolute abundance of aphids per volume of air, including aphids which are not in ‘alighting mode’ [55],[56]. In contrast, YPTs are specifically catching individuals in the ‘alighting mode’ [57],[58]. This may be an advantage in use of YPTs for monitoring virus vectors, because the estimated landing rate on plants is the important factor in such context. It has also been suggested that the two types of traps discriminate aphids based on colour preferences [59]. Finally, aphid species feeding on dicots have been found to be biased towards YPTs, whereas aphids feeding on grasses and sedges are caught in higher relative numbers with a suction trap [60]. Our results seem to support these previously reported results.

The total number of aphids caught by the suction trap ranged from 60 to 1100 individuals per year, revealing a low overall abundance compared to other seed potato production areas, e.g., in central Europe [61]. Furthermore, the long term suction trap data obtained over a decade revealed a recurring three-year cyclic pattern in overall abundance of aphids. Similar recurring patterns, but with a biennial interval, have been found in the northern seed potato growing areas in New Brunswick in Canada [62],[63] and in The Netherlands in 1936–1954 [64]. The patterns have been explained by a cyclic prey-predator effect in which large, early aphid populations enhance the build-up of predator populations, which in turn reduces aphid populations prior to migration of aphids to winter hosts and hence leads to smaller aphid populations in the following year. The underlying cause of the observed triennial pattern in this study might be attributable to a similar mechanism which takes three years instead of two due to the shorter growing season and slower population build-up in Tyrnävä-Liminka area. Both locations with the observed biennial cycle in Canada and the Netherlands are located much further south (ca. 46° N) than Tyrnävä-Liminka area (64° N).

Suction traps and YPTs are regularly used for monitoring aphid abundance. The 12.2 m high Rothamsted suction trap provides information about the composition and long distance movement of aphid taxa in large areas and helps to recognise an overall increase in the risk of pest problems and virus transmission [65],[66]. Local differences may be important for aphid control programmes where monitoring of aphids in the field informs the threshold risk level of virus transmission and the moment to begin measures for control of aphids. Local data are also needed for construction of empirical epidemiological models [67]. We used YPT data for the phenological analysis, because it offered a larger total catch and data base than the suction trap (56,040 vs. 2488 aphid individuals, respectively), suggesting that YPTs are advantageous for determination of phenological patterns.

This study suggests that DNA barcoding is a useful tool for supplementing morphological identification of aphid species, such as *Aphis gossypii* and *Aphis frangulae*, which are closely similar in morphology but differ in their impact on agriculture. Our study provided new knowledge on the occurrence and phenology of aphid species, which can inform enhanced pest management and risk assessment schemes in crops such as potatoes and cereals in northern agricultural regions. Measures for the control of plant viruses are often dependent on the temporal occurrence of vectors. For example, mulching is more effective against the spread of viruses when the vector flight occurs early in the season [8]. The revealed triennial cycle in abundance of aphids may also be utilised for annual risk assessment. Taken together, this study provides a comprehensive picture of aphid fauna in one of the northernmost intensive crop production areas of the world and indicates that the aphid species occurring there form distinguishable groups according to their phenology (timing of flight activity). Further studies can build on our results to establish whether the observed phenology patterns are more widespread geographically. In addition, the data presented here can serve as a baseline for future comparisons of the aphid fauna and phenology in the changing climate [68].

### Sequence Data

BOLD Systems database accession numbers AFNF001-12 to AFNF004-12 and AFNF006-12 to AFNF0034-12 (*COI* barcode sequences of 33 aphid species), AFNF005-12 and AFNF0035-12 to AFNF0039-12 (six bar-coded individuals of *Aphis gossypii*).

## Supporting Information

Figure S1 **Analysis of** ***Aphis frangulae*** **/** ***gossypii*** **complex based on** ***COI*** **barcode sequences using neighbour-joining analysis.** Genetic distances (nucleotide substitutions) are indicated with a scale bar. The colours of accession numbers indicate the following: Black: *Aphis gossypii* representing a broad geographical area; Red: *Aphis gossypii* from Northern Finland; Blue: *Aphis frangulae*; Green: *Aphis fabae* used as an outgroup. Bootstrap values of 1000 replicates are shown. Further information is available in Barcode of Life Data Systems homepage (BOLD; www.boldsystems.org).
(PDF)

Table S1 **Partial sequences of the cytochrome c oxidase I (** ***COI*** **) genes of aphids determined in this study.**
(PDF)
