# Supplementary figures and images for: Seasonal Phenology and Species Composition of the Aphid Fauna in a Northern Crop Production Area
Source: PLoS One. 2013 Aug 13;8(8):e71030. doi: 10.1371/journal.pone.0071030 (PMC3742763; doi:10.1371/journal.pone.0071030)

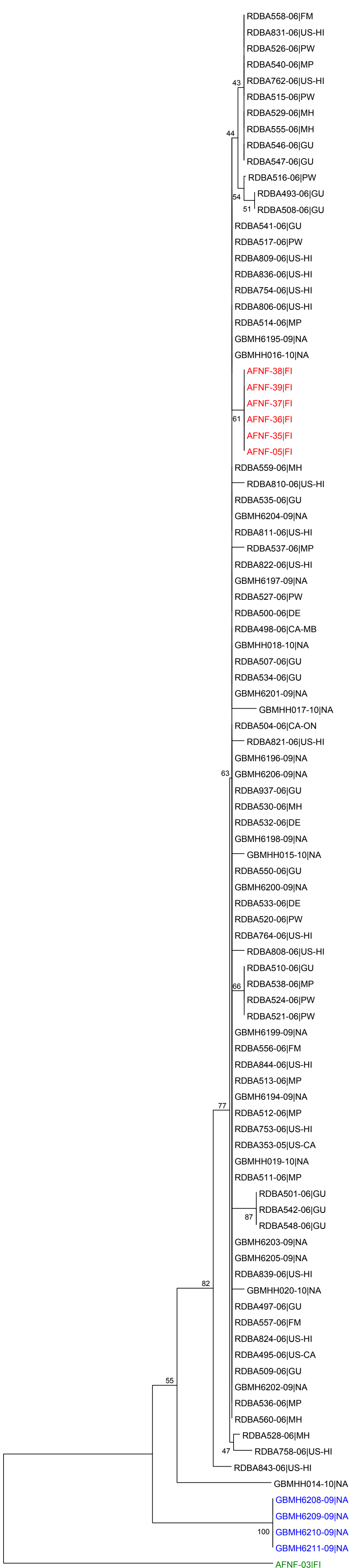

0.005

Supplement: Figure S1 — Analysis of Aphis frangulae / gossypii complex based on COI barcode sequences using neighbour-joining analysis. Genetic distances (nucleotide substitutions) are indicated with a scale bar. The colours of accession numbers indicate the following: Black: Aphis gossypii representing a broad geographical area; Red: Aphis gossypii from Northern Finland; Blue: Aphis frangulae; Green: Aphis fabae used as an outgroup. Bootstrap values of 1000 replicates are shown. Further information is available in Barcode of Life Data Systems homepage (BOLD; www.boldsystems.org). (PDF) [file pone.0071030.s001.pdf]
