# Supplementary material for: Seasonal Phenology and Species Composition of the Aphid Fauna in a Northern Crop Production Area
Source: PLoS One. 2013 Aug 13;8(8):e71030. doi: 10.1371/journal.pone.0071030 (PMC3742763; doi:10.1371/journal.pone.0071030)
Supplement: Table S1 — Partial sequences of the cytochrome c oxidase I ( COI ) genes of aphids determined in this study. (PDF) [file pone.0071030.s002.pdf]

Table S1. Partial sequences of the cytochrome c oxidase I (*COI*) genes of aphids determined in this study. For additional information, search the Barcode of Life Data (BOLD) Systems database at www.boldsystems.org - Public Data using AFNF as the keyword.

**Accession number**	**Species**	**Partial *COI* sequence**
AFNF001-12	*Acyrthosiphon pisum*	AACTTTATACTTTTTATTTGGTATTTGATCAGGTATAATTGGATCTTCACTTAGAATTCTAATTCGTTTAGAATT<br>AAGTCAAATTAATTCTATTATTAACAATAATCAATTATATAATGTAATTGTTACAATTCATGCTTTTATTATAAT<br>TTTTTTTATAACTATACCAATTGTAATTGGTGGATTTGGAAATTGATTAATTCCTATAATAATAGGATGTCCTG<br>ATATATCATTTCCTCGCTTAAATAATATTAGATTTTGATTATTACCTCCTTCATTAATAATAATAATTTGCAGTT<br>TCTTAATTAATAATGGAACAGGAACAGGATGAACTATTTATCCACCTTTATCAAATAATATTGCACATAATAA<br>CATTTCAGTTGATTTAACTATTTTTTCTTTACACCTAGCAGGAATTTCATCAATTTTAGGAGCAATTAATTTTAT<br>TTGTACAATTCTTAATATAATACCTAATAACATAAAATTAAATCAAATTCCACTTTTCCCTTGATCAATTTTAAT<br>TACAGCTATCTTATTAATTTTATCTTTACCAGTTTTAGCTGGTGCTATTACAATATTATTAACTGATCGAAACTT<br>AAATACATCATTTTTTGATCCAGCAGGAGGAGGAGATCCTATTTTATACCAACATTTATTT
AFNF002-12	*Amphorophora rubi*	AACTTTATACTTTTTATTTGGTATCTGATCAGGTATAATTGGATCATCACTTAGAATCTTAATTCGACTAGAAT<br>TAAGACAAATTAATTCAATTATTAATAACAATCAATTATATAATGTAATTGTAACAATTCATGCTTTTATTATA<br>ATTTTTTTTATAACAATACCAATTGTAATTGGTGGTTTTGGAAATTGATTAATTCCTATAATAATAGGATGTCC<br>TGATATATCATTTCCACGTCTAAATAATATTAGATTTTGATTACTACCCCCATCATTAATAATAATAATTTGTA<br>GTTTTTTAATTAATAATGGTACAGGAACAGGATGAACTATTTATCCACCTTTATCAAATAATATTGCACATAA<br>TAATATTTCAGTTGATTTAACTATTTTTTCTTTACATTTAGCAGGAATTTCATCAATTTTAGGAGCAATTAATTT<br>TATTTGTACTATTTTAAATATAATACCAAACAATATAAAATTAAATCAAATTCCTTTATTCCCTTGATCAATTTT<br>AATTACAGCTATTTTATTAATTTTATCCTTACCTGTATTAGCAGGTGCTATTACAATATTATTAACTGATCGTA<br>ATTTAAATACATCATTTTTTGATCCAGCAGGAGGAGGAGATCCTATTTTATATCAACATTTATTT
AFNF003-12	*Aphis fabae*	AACTTTATATTTTTTATTTGGTATTTGATCAGGTATAATTGGATCTTCACTTAGAATTTTAATTCGATTAGAATT<br>AAGACAAATTAATTCAATTATTAATAATAATCAACTATATAATGTAATTGTTACAATTCATGCTTTTATTATAA<br>TTTTTTTTATAACTATACCAATTGTAATTGGAGGTTTTGGAAATTGATTAATTCCTATAATAATAGGATGCCCA<br>GACATATCTTTCCCACGATTAAATAATATTAGATTTTGATTATTACCACCATCACTAATAATAATAATTTGTAG<br>ATTTATAATTAATAACGGAACAGGAACAGGATGAACTATTTATCCACCCCTATCAAATAATATTGCCCATAAT<br>AATATTTCAGTTGATTTAACTATTTTTTCCCTTCATCTAGCAGGTATTTCATCAATTTTAGGAGCAATTAATTTT<br>GTTTGCACTATTTTAAATATAATACCTAATAATATAAAATTAAATCAAATTCCCCTATTTCCATGATCAATCTT<br>AATTACAGCCATATTATTAATTTTATCCTTACCAGTTTTAGCTGGTGCTATTACTATATTATTAACTGATCGAA<br>ATTTAAATACATCATTTTTTGACCCAGCAGGAGGAGGAGATCCAATTCTTTATCAACATTTATTT
AFNF004-12	*Aphis pomi*	AACTTTATATTTTTTATTTGGTATTTGATCAGGAATAATTGGATCTTCTCTTAGAATTTTAATTCGATTAGAATT<br>AAGTCAAATTAATTCAATTATCAACAATAACCAATTATATAATGTAATTGTTACAATTCATGCTTTTATTATAA<br>TTTTTTTTATAACTATACCAATTGTAATTGGTGGATTTGGAAATTGATTAATCCCTATAATAATAGGGTGCCCA<br>GATATATCTTTCCCACGATTAAATAATATTAGATTCTGATTATTACCGCCTTCACTAATAATAATAATTTGTAG<br>ATTTATAATTAATAATGGAACAGGAACAGGATGAACAATTTATCCACCCTTATCAAATAATATTGCCCATAAT<br>AATATTTCAGTTGATTTAACCATTTTTTCTCTTCACCTAGCAGGTATTTCATCAATTTTAGGAGCAATTAATTTT<br>ATTTGCACAATCCTCAATATAATACCAAATAATATAAAATTAAATCAAATCCCACTATTCCCATGATCAATCCT<br>AATTACAGCTATATTATTAATTTTATCTTTACCAGTTTTAGCTGGTGCTATTACGATACTATTAACTGACCGAA<br>ATTTAAATACATCATTTTTTGATCCAGCAGGAGGAGGGGATCCAATTCTTTACCAACACTTATTC
AFNF005-12	*Aphis gossypii*	AACTTTATATTTTTTATTTGGTATTTGATCAGGTATAATTGGTTCTTCTCTTAGAATTTTAATCCGATTAGAATT<br>AAGTCAAATTAATTCAATTATTAATAATAATCAATTATATAATGTAATTATTACAATTCATGCTTTTATTATAAT<br>TTTTTTTATAACTATACCAATCGTTATTGGAGGTTTTGGAAATTGATTAATTCCTATAATAATAGGATGTCCAG<br>ATATATCTTTTCCACGACTAAATAATATTAGATTCTGATTATTACCACCCTCATTAATAATAATAATTTGCAGA<br>TTTATAATTAATAACGGAACAGGAACAGGATGAACTATTTATCCACCTTTATCAAATAATATTGCTCATAATA<br>ATATTTCAGTAGACTTAACTATTTTTTCCCTACATTTAGCAGGTATCTCATCAATTTTAGGAGCAATTAATTTC<br>ATCTGTACTATCTTAAATATAATACCTAATAATATAAAATTAAATCAAATTCCTCTATTTCCATGATCAATTTTA<br>ATTACAGCTATATTATTAATTTTATCCTTACCTGTATTAGCTGGTGCTATTACTATATTATTAACAGATCGAAA<br>TTTAAATACATCATTTTTTGATCCAGCAGGTGGGGGAGACCCTATTCTTTATCAACATTTATTT
AFNF006-12	*Aphis idaei*	AACTTTATATTTTTTATTTGGTATTTGATCAGGTATAATTGGATCTTCATTAAGAATTTTAATTCGATTAGAAT<br>TAAGTCAAATTAATTCAATTATTAATAATAATCAATTATATAATGTAATTGTTACAATTCATGCTTTTATTATA<br>ATTTTTTTTATAACTATACCAATTGTAATTGGAGGCTTTGGAAATTGATTAATCCCTATAATAATAGGTTGTCC<br>AGATATATCTTTTCCACGACTAAATAATATCAGATTCTGATTATTACCACCTTCATTAATAATAATAATTTCCA<br>GATTTATAATTAATAACGGAACAGGAACAGGATGAACTATTTATCCACCTTTATCAAATAATATTGCTCATAA<br>TAATATCTCAGTTGACTTAACTATTTTTTCTCTTCATTTAGCAGGTATTTCATCAATTTTAGGAGCAATTAATTT<br>TATTTGTACTATCTTAAATATAATACCCAATAATATAAAATTAAATCAAATTCCCCTATTCCCATGATCAATCTT<br>AATCACAGCTATATTATTAATTTTATCTTTACCAGTTTTAGCTGGTGCTATTACTATATTATTAACAGATCGAA<br>ATCTAAACACATCATTTTTTGATCCAGCCGGTGGAGGAGATCCTATTCTTTATCAACATTTATTC

AFNF007-12	*Aulacorthum solani*	AACTTTATATTTTTTATTTGGTATTTGATCAGGTATAATTGGATCATCCCTTAGAATTTTAATTCGTCTAGAAC<br>TAAGACAAATTAATTCTATTATTAACAATAATCAATTATATAATGTAATTGTTACAATTCACGCTTTTATTATA<br>ATTTTTTTTATAACAATACCAATTGTAATTGGTGGGTTTGGAAATTGATTAATCCCTATAATAATAGGATGTC<br>CTGATATATCATTTCCACGTTTAAATAATATTAGATTTTGATTATTACCTCCTTCATTAATAATAATAATTTGTA<br>GTTTCCTAATTAATAATGGAACAGGAACAGGATGAACTATTTATCCACCTTTATCAAATAATATTGCACATAA<br>TAATATTTCAGTTGACTTAACTATTTTTTCCCTACATTTAGCAGGAATTTCATCAATTTTAGGAGCAATTAATTT<br>TATTTGTACAATTCTTAATATAATACCAAACAATATAAAATTAAATCAAATTCCTCTTTTCCCTTGATCAATTTT<br>AATTACAGCTATTTTATTAATTTTATCTTTACCAGTATTAGCTGGTGCTATTACAATATTATTAACTGATCGTA<br>ATCTAAATACATCATTTTTTGATCCAGCCGGAGGAGGAGATCCAATTTTATATCAACATTTATTT
AFNF008-12	*Calaphis flava*	AACTTTATACTTTTTATTTGGAATTTGATCAGGGATGATTGGATCATCTTTAAGAATTTTAATTCGACTAGAAT<br>TAAGACAAATTAATTCAATTATTAACAATAATCAATTATATAATGTTATTGTAACTATTCATGCATTTATTATA<br>ATTTTTTTTATAACTATACCAATTGTAATTGGAGGATTTGGAAATTGATTAATTCCTATAATAATAGGATCACC<br>TGATATATCTTTCCCTCGACTTAATAATATTAGATTTTGATTACTACCCCCTTCTTTAATAATAATAATTAGAAG<br>ATTTTTAATTAATAATGGAACAGGAACAGGATGAACAATTTACCCTCCTTTATCAAACAATATTGCCCATAAT<br>AATATTTCAGTAGATTTAACAATTTTTTCTTTACATTTAGCAGGAATTTCATCAATCTTAGGAGCTATTAATTTT<br>ATTTGTACAATTTTAAATATAATGCCTAATAATATAAAATTAAATCAAATTCCATTATTCCCATGATCTATCTT<br>AATTACAGCAATTTTATTAATCCTATCTCTCCCAGTTTTAGCTGGAGCTATTACTATATTATTAACTGATCGAA<br>ATTTAAATACATCATTTTTTGACCCTTCCGGAGGAGGGGACCCAATCCTTTACCAACATTTATTC
AFNF009-12	*Calaphis betulicola*	AACTTTATATTTTTTATTTGGAATCTGATCAGGAATGATTGGGTCATCATTAAGAATTTTAATTCGATTAGAAT<br>TAAGTCAAATTAATTCAATTATTAATAATAATCAATTATATAATGTTATTGTAACTATTCATGCATTTATTATA<br>ATTTTCTTTATAACAATACCAATTGTAATTGGGGGATTTGGAAATTGACTAATTCCTTTAATAATAGGGTCAC<br>CTGATATATCATTTCCTCGACTTAATAATATTAGATTTTGATTATTACCCCCCTCTTTAATAATAATAATTAGAA<br>GATTTTTAATTAATAATGGAACAGGAACTGGTTGAACAATTTACCCGCCCCTATCAAATAATATTGCACACAA<br>TAATATCTCAGTTGATTTAACAATTTTTTCCCTACATCTAGCCGGAATTTCATCAATTTTAGGTGCAATTAATTT<br>CATTTGTACAATTTTAAATATAATACCTAATAATATAAAATTAAATCAAATTCCTTTATTCCCATGATCAATTTT<br>AATTACAGCAATTCTATTAATTCTATCTCTCCCAGTTTTAGCAGGAGCTATTACTATACTTTTAACTGATCGAA<br>ATCTTAATACATCATTCTTTGATCCTTCCGGGGGAGGTGATCCAATTCTTTACCAACACTTATTT
AFNF010-12	*Capitophorus hippophaes*	AACTTTATACTTTTTATTTGGTATCTGATCAGGTATAATTGGATCTTCACTAAGAATTTTAATTCGTTTAGAAT<br>TAAGCCAAATTAATTCAATTATTAATAATAATCAACTATACAATGTAATTGTTACAATTCATGCTTTCATTATA<br>ATTTTCTTTATGACTATACCAATTGTAATTGGTGGGTTTGGAAATTGACTAATCCCAATAATAATAGGATGTC<br>CTGATATATCATTCCCACGACTAAATAATATTAGATTTTGATTATTACCACCTTCACTAATAATAATAATCTGT<br>AGATTCTTAATTAATAATGGAACAGGAACAGGATGAACTATTTATCCCCCCTTATCTAATAATATTGCGCATA<br>ATAATATTTCAGTAGATTTAACTATTTTCTCTCTACATTTAGCAGGAATCTCATCAATTTTAGGAGCAATTAAT<br>TTTATTTGTACAATTCTAAATATAATACCTTATAATATAAAACTAAATCAAATTCCTCTTTTCCCATGATCAATT<br>TTAATCACAGCAATCTTATTAATTTTATCTTTACCAGTTCTAGCAGGTGCTATTACAATATTATTAACTGATCG<br>AAATTTAAATACTTCATTTTTTGATCCAGCAGGAGGAGGAGATCCAATTCTATATCAACATTTATTT
AFNF011-12	*Cavariella theobaldi*	AACTTTATACTTTTTATTTGGTATTTGATCAGGTATAATTGGATCTTCTCTTAGAATTTTAATTCGATTAGAATT<br>AAGTCAAATTAACTCCATTATTAACAATAATCAATTATATAATGTAATTGTTACTATTCATGCTTTTATTATAAT<br>TTTCTTTATAACTATACCAATCGTAATTGGTGGTTTTGGAAATTGATTAATTCCTATAATAATAGGATGCCCTG<br>ATATGTCATTTCCACGATTAAATAATATCAGTTTTTGATTATTACCACCCTCACTAATAATAATAATTTGTAGT<br>TTTTTAATTAATAACGGAACAGGAACAGGATGAACTATTTACCCACCACTATCTAATAATATTGCACATAACA<br>ATATCTCAGTTGATTTAACTATTTTTTCATTACATTTAGCCGGAGTTTCATCAATTTTAGGAGCAATTAACTTT<br>ATTTGCACTATTCTAAATATAATACCTTATAATATAAAACTTAATCAAATTCCATTATTCCCATGATCAATCTTA<br>ATTACAGCTATTTTATTAATCTTATCATTACCCGTTTTAGCAGGAGCTATTACAATATTATTAACTGACCGAAA<br>TTTAAATACATCATTTTTTGATCCAGCAGGAGGAGGAGATCCAATTTTATACCAACATTTATTT
AFNF012-12	*Cavariella pastinacae*	AACTTTATACTTTTTATTTGGTATTTGATCAGGTATAATTGGATCTTCCCTTAGAATTTTAATTCGATTAGAATT<br>AAGTCAAATTAACTCTATTATTAACAATAATCAATTATATAATGTAATTGTTACTATTCATGCTTTTATTATAAT<br>TTTCTTCATAACTATACCAATTGTAATTGGTGGTTTTGGAAATTGATTAATTCCTATAATAATAGGATGCCCTG<br>ATATATCATTTCCACGATTAAATAATATTAGTTTTTGATTATTACCACCTTCATTAATAATAATAATTTGCAGTT<br>TTTTAATTAATAACGGAACAGGAACAGGATGAACTATTTACCCACCATTATCTAACAATATTGCACATAATAA<br>TATCTCAGTCGATTTAACTATTTTTTCACTACATTTAGCAGGAATCTCATCAATTTTAGGAGCAATTAATTTTA<br>TTTGTACTATTTTAAATATAATACCTTATAATATAAAACTTAATCAAATTCCTTTATTTCCATGATCAATTTTAA<br>TTACAGCTATTCTATTAATCTTATCACTACCTGTTTTAGCAGGAGCTATTACAATATTATTAACAGATCGAAAT<br>TTAAATACATCATTTTTTGACCCAGCAGGAGGAGGAGATCCAATTTTATATCAACATTTATTT
AFNF013-12	*Cavariella aegopodii*	AACTTTATACTTTCTATTTGGTATTTGATCAGGTATAATTGGATCTTCTCTTAGAATTTTAATTCGATTAGAATT<br>AAGTCAAATTAACTCTATTATTAATAATAATCAATTATACAACGTAATTGTTACTATTCATGCTTTTATTATAAT<br>TTTTTTTTATAACTATACCAATTGTAATTGGTGGTTTTGGAAATTGATTAATTCCTATAATAATAGGATGCCCTG<br>ACATATCATTTCCACGATTAAATAACATTAGTTTTTGATTATTACCACCTTCATTAATAATAATAATTAGTAGT<br>TTTTTAATTAATAACGGAACAGGAACAGGATGAACTATTTATCCACCACTATCTAATAACATCGCACATAATA<br>ATATTTCAGTTGATTTAACTATCTTCTCATTACATTTAGCAGGAATTTCATCAATTTTAGGAGCAATTAATTTT<br>ATTTGCACTATCTTGAATATAATACCTTATAATATAAAACTTAATCAAATTCCTCTATTTCCATGATCAATTTTA<br>ATTACAGCTATTTTATTAATTTTATCATTACCTGTTTTAGCAGGAGCTATTACAATATTACTAACTGACCGAAA<br>TTTAAATACATCATTTTTTGATCCAGCAGGAGGAGGAGATCCAATTTTATATCAACATTTATTT
AFNF014-12	*Cavariella konoi*	AACTTTATATTTCTTATTTGGTGTATGATCAGGTATAATTGGATCTTCTCTTAGAATTTTAATTCGATTAGAAT<br>TAAGACAAATTAATTCAATTATTAATAATAATCAATTATATAATGTAATTGTTACTATTCATGCTTTTATTATA<br>ATTTTTTTTATAACTATACCAATTGTAATTGGAGGATTTGGAAACTGATTAATTCCTATAATAATAGGATGTCC<br>TGATATATCATTTCCACGATTAAATAATATTAGATTTTGATTATTACCACCTTCATTAATAATAATAATTTGCA<br>GTTTTTTAATTAATAATGGAACAGGAACAGGATGAACTATTTATCCACCTTTATCTAATAATATTGCACATAA<br>TAATATTTCAGTTGATTTAACTATTTTTTCATTACATTTAGCAGGAATCTCATCAATTTTAGGAGCAATCAACT<br>TTATTTGTACTATTTTAAATATAATACCTTATAATATAAAATTAAATCAAATTCCTTTATTTCCATGATCAATCT<br>TAATTACAGCTATTTTATTAATTTTATCTCTACCTGTATTAGCAGGTGCAATTACAATATTATTAACTGATCGA<br>AATTTAAATACATCATTTTTTGATCCAGCAGGAGGGGGTGATCCAATTTTATATCAACATTTATTT

AFNF015-12	*Cryptomyzus galeopsidis*	AACTTTATATTTTTTATTTGGTATTTGATCAGGTATAATTGGATCTTCTCTTAGAATTTTAATTCGTTTAGAATT<br>AAGACAAATTAATTCAATTATTAATAATAATCAATTGTATAATGTAATTGTAACAATTCATGCTTTCATTATAA<br>TTTTTTTTATAACAATACCAATTGTTATTGGTGGTTTTGGAAATTGATTAATTCCTATAATAATAGGATGTCCA<br>GATATATCATTTCCACGTTTAAATAATATTAGATTTTGATTATTACCACCATCATTAATAATAATAATTTGTAG<br>TTTTTTAATTAATAATGGAACAGGAACAGGATGAACTATTTACCCACCTTTATCAAATAATATTGCACATAAT<br>AATATTTCAGTTGATTTAACTATTTTTTCTTTACATTTAGCAGGAATTTCATCAATTTTAGGAGCAATTAATTTT<br>ATTTGTACAATTTTAAATATAATACCAAATAATATAAAATTAAATCAAATTCCTCTTTTTCCTTGATCAATTTTA<br>ATTACAGCTATTTTATTAATTTTATCTTTACCAGTATTAGCTGGTGCCATTACAATACTATTAACTGATCGTAA<br>TTTAAATACATCATTTTTTGATCCAGCAGGAGGAGGAGATCCTATTTTATATCAACATTTATTT
AFNF016-12	*Cryptomyzus stachydis*	AACTTTATATTTCCTATTTGGTATTTGATCAGGTATAATTGGATCATCTCTTAGAATTTTAATTCGTTTAGAATT<br>AAGTCAAATTAATTCAATTATTAATAACAATCAATTATATAATGTAATTGTAACAATTCATGCTTTTATTATAA<br>TTTTTTTTATAACAATACCAATTGTTATTGGTGGATTTGGAAATTGATTAATCCCTATAATAATAGGATGCCCT<br>GATATATCATTCCCACGATTAAATAATATTAGATTTTGATTATTACCACCATCATTAATAATAATAATTTGTAG<br>TTTTTTAATTAATAACGGAACAGGAACAGGATGAACTATTTACCCACCTTTATCAAATAATATTGCACATAAT<br>AATATTTCAGTTGATTTAACTATTTTTTCTTTACATTTAGCAGGAATCTCATCAATCTTAGGAGCAATTAACTTT<br>ATTTGTACAATTTTAAATATAATACCCAATAATATAAAACTAAATCAAATTCCTCTTTTCCCTTGATCAATTTTA<br>ATTACAGCTATTTTATTAATTTTATCCTTACCAGTATTAGCTGGTGCTATTACAATATTATTAACTGATCGTAAT<br>TTAAATACATCATTTTTTGATCCAGCAGGAGGAGGTGATCCTATTTTATATCAACATCTATTT
AFNF017-12	*Elatobium abietinum*	AACTTTATACTTTTTATTTGGTATTTGATCAGGTATAATTGGATCTTCACTTAGAATCTTAATTCGACTAGAAT<br>TAAGTCAAATTAATTCAATTATTAATAATAATCAATTATATAATGTAATTGTAACAATTCATGCTTTTATTATA<br>ATTTTCTTTATAACTATACCAATTGTAATTGGTGGCTTTGGTAATTGATTAATTCCTATAATAATAGGATGCCC<br>AGACATATCTTTCCCACGATTAAATAATATTAGTTTTTGACTTTTACCCCCTTCTTTAATAATAATAATTTCTAG<br>ATTTTTAATTAATAATGGAACAGGAACAGGTTGAACTATTTACCCACCTTTATCAAACAATATTGCACATAAT<br>AATATTTCAGTTGATCTAACCATTTTTTCTTTACATTTAGCTGGAATTTCATCAATTTTAGGAGCAATTAATTTT<br>ATTTGTACAATTTTAAATATAATACCAAATAATATAAAATTAAATCAAATTCCATTATTTCCTTGATCAATCTTA<br>ATTACAGCTATTTTATTAATCCTATCCCTACCTGTATTAGCAGGTGCTATTACAATACTTTTAACTGATCGAAA<br>TTTAAATACATCATTTTTTGATCCAGCTGGAGGAGGAGATCCAATTTTATATCAACATTTATTT
AFNF018-12	*Hayhurstia atriplicis*	AACTTTATATTTTTTATTCGGAATTTGATCAGGTATAATTGGATCATCTCTTAGAATTTTAATTCGATTAGAAT<br>TAAGTCAAATTAATTCAATTATTAACAACAATCAATTATATAATGTAATTGTTACAATTCACGCTTTCATTATA<br>ATTTTTTTTATAACAATACCAATTGTTATTGGAGGATTTGGAAATTGATTAATTCCTATAATAATAGGATGTCC<br>TGATATATCATTTCCTCGATTAAATAATATTAGATTTTGATTACTCCCACCATCATTAATAATAATAATTTGTA<br>GTTTTTTAATTAACAATGGAACAGGAACAGGATGAACTATTTACCCACCCTTATCAAATAATATTGCCCATAA<br>TAATATTTCAGTTGATTTAACTATTTTTTCTTTACATTTAGCAGGAATTTCATCAATTTTAGGAGCAATTAATTT<br>TATTTGTACAATTTTAAATATAATACCTAATAATATAAAATTAAATCAAATTCCTCTTTTCCCATGATCAATCTT<br>AATTACAGCTATTTTATTAATTTTATCTCTCCCAGTATTAGCTGGTGCTATTACAATATTATTAACTGATCGAA<br>ATTTAAATACTTCATTTTTTGATCCAGCAGGAGGGGGAGATCCTATTTTATATCAACATCTATTT
AFNF019-12	*Hyalopterus pruni*	AACTTTATATTTTTTATTTGGTATTTGATCAGGTATAATTGGATCTTCTCTTAGAATATTAATTCGATTAGAAC<br>TTAGACAAATTAATTCAATTATTAATAATAATCAACTTTACAATGTGATTGTTACAATCCATGCTTTCATTATA<br>ATTTTCTTTATAACTATACCAATTGTAATTGGTGGATTTGGAAATTGATTAATTCCTATAATAATAGGATGTCC<br>TGATATATCTTTCCCTCGATTAAATAATATTAGATTTTGATTACTCCCCCCTTCCTTAATAATAATAATCTGTAG<br>ATTTATAATTAATAACGGAACAGGAACAGGATGAACAATTTATCCACCATTATCTAATAATATTGCACATAAT<br>AATATTTCAGTTGATTTAACAATTTTTTCACTTCATTTAGCAGGAATCTCATCAATTTTAGGAGCAATTAATTT<br>TATTTGCACAATTTTAAATATAATACCTAATAACATAAAATTAAATCAAATTCCTTTATTTCCATGATCAATTTT<br>AATTACAGCTACCTTATTAATTTTATCACTCCCAGTTTTAGCTGGAGCTATTACTATATTATTAACTGATCGTA<br>ATTTAAATACATCATTTTTTGATCCTGCTGGAGGAGGAGATCCAATTCTTTACCAACACTTATTC
AFNF020-12	*Hyperomyzus lactucae*	AACTTTATATTTTTTATTTGGTATTTGATCAGGTATAATTGGCTCATCACTTAGAATTTTAATTCGATTAGAAT<br>TAAGACAAATTAATTCAATTATTAATAATAACCAATTATATAATGTAATTGTTACAATTCATGCTTTCATTATA<br>ATTTTTTTTATAACAATACCAATTGTTATTGGTGGTTTTGGAAACTGATTAATTCCTATAATAATAGGATGTCC<br>AGATATATCATTTCCACGTTTAAATAATATTAGATTTTGATTACTACCTCCATCATTAATAATAATAATTTGCA<br>GATTTTTAATTAATAATGGAACAGGAACAGGATGAACTATTTATCCACCTTTATCAAATAATATTGCACATAA<br>TAATATTTCAGTTGACTTAACTATTTTTTCCTTACATTTAGCAGGAATCTCATCAATTTTAGGAGCAATTAATTT<br>TATTTGTACAATTTTAAATATAATACCTAATAATATAAAATTAAATCAGATTCCTCTTTTTCCTTGATCAATTTT<br>AATTACAGCTATTTTATTAATTTTATCTTTACCAGTACTAGCTGGTGCTATTACAATATTATTAACTGATCGTA<br>ATTTAAATACCTCATTTTTTGATCCAGCAGGAGGAGGAGATCCTATTTTGTATCAACATTTATTT
AFNF021-12	*Hyperomyzus rhinanthi*	AACTTTATACTTTTTATTTGGTATTTGATCAGGTATAATTGGATCATCTCTTAGAATTTTAATCCGTCTAGAAT<br>TAAGTCAAATTAACTCAATTATTAATAATAATCAATTATATAATGTAATTGTAACAATTCACGCTTTTATTATA<br>ATTTTTTTTATAACAATACCAATTGTTATTGGTGGTTTCGGAAACTGGTTAATTCCTATAATAATAGGATGTCC<br>TGATATATCATTCCCACGTTTAAATAATATTAGATTTTGATTATTACCACCATCATTAATAATAATAATTTGTA<br>GTTTTTTAATTAATAATGGAACAGGAACAGGATGAACTATCTATCCCCCTTTATCAAATAATATTGCACACAA<br>TAATATTTCAGTTGATTTAACTATTTTTTCATTACATTTAGCAGGAATCTCATCAATCTTAGGAGCAATTAATT<br>TTATTTGCACAATTTTAAATATAATACCAAATAATATAAAATTAAATCAAATCCCTCTTTTCCCTTGATCAATTT<br>TAATTACAGCTATTTTATTAATTTTATCATTACCAGTACTAGCTGGTGCTATTACAATATTATTAACTGATCGT<br>AATTTAAATACATCATTTTTTGATCCAGCAGGAGGAGGAGATCCAATCTTATACCAACATTTATTT
AFNF022-12	*Macrosiphum rosae*	AACTTTATACTTTTTATTTGGTATTTGATCAGGTATAATTGGATCATCTCTTAGAATTTTAATTCGATTAGAAT<br>TAAGACAAATTAATTCTATTATTAATAATAATCAATTATATAATGTAATTGTTACAATTCATGCTTTTATTATA<br>ATTTTTTTTATAACTATACCAATTGTAATTGGAGGATTTGGAAATTGATTAATTCCTATAATAATAGGATGCCC<br>TGATATATCATTTCCACGTTTAAATAATATTAGATTTTGATTATTACCTCCATCATTAATAATAATAATTTGTAG<br>ATTTTTAATTAATAACGGTACAGGAACAGGATGAACAATTTATCCACCTTTATCAAACAATATTGCACACAAT<br>AATATTTCAGTTGATTTAACTATTTTTTCTCTGCATTTAGCAGGAATTTCATCAATCTTAGGAGCAATTAACTT<br>TATTTGTACAATTCTTAATATAATACCAAATAATTTAAAACTTAATCAAATTCCTCTCTTTCCTTGATCAATTTT<br>AATTACAGCTATTTTACTAATTTTATCTTTACCAGTTTTAGCCGGTGCTATTACAATATTACTAACTGATCGTA<br>ATTTAAATACATCATTTTTTGATCCAGCAGGAGGAGGAGACCCTATTTTATATCAACATTTATTT

AFNF023-12	*Macrosiphum euphorbiae*	AACTTTATACTTTTTATTTGGTATTTGATCAGGTATAATTGGATCATCTCTTAGAATTTTGATTCGATTAGAAT<br>TAAGACAAATTAATTCTATTATTAATAATAATCAATTATATAATGTAATTGTTACAATTCATGCTTTTATTATA<br>ATTTTTTTTATAACTATACCAATTGTAATTGGTGGATTTGGAAATTGATTAATTCCTATAATAATAGGATGTCC<br>TGATATATCATTTCCACGTTTAAATAATATTAGATTTTGATTACTACCTCCATCATTAATAATAATAATTTGTA<br>GATTTTTAATTAATAATGGAACAGGAACAGGATGAACAATTTATCCCCCTTTATCAAACAATATTGCACATAA<br>TAACATTTCAGTTGATTTAACTATTTTTTCTTTACATCTAGCAGGAATCTCATCAATTTTAGGAGCAATTAATTT<br>TATTTGTACAATTCTTAATATAATACCAAACAATATAAAATTAAATCAAATTCCTCTTTTTCCTTGATCAATTTT<br>AATTACAGCTATTTTACTAATTTTATCTTTACCAGTTTTAGCTGGTGCCATTACAATACTTTTAACTGATCGTAA<br>TTTAAATACATCATTTTTTGATCCAGCAGGAGGAGGAGATCCTATTTTATATCAACATTTATTT
AFNF024-12	*Megoura viciae*	AACTTTATACTTTTTATTTGGTATTTGATCAGGTATAATTGGATCATCACTTAGAATTTTAATTCGTCTAGAAT<br>TAAGACAAATTAATTCAATTATTAATAATAATCAATTATATAATGTAATTGTAACAATTCATGCTTTTATTATA<br>ATTTTTTTTATAACTATACCTATTGTAATTGGTGGTTTTGGAAATTGATTAATTCCTATAATAATAGGATGTCC<br>TGACATATCATTTCCACGTTTAAATAATATTAGATTTTGACTATTACCACCATCATTAATAATAATAATTTGTA<br>GTTTTTTAATTAATAATGGTACAGGAACAGGATGAACTATTTATCCACCTTTATCAAATAATATTGCACATAA<br>TAATATTTCAGTTGATTTAACTATTTTTTCATTACATTTAGCAGGAATTTCATCAATTTTAGGAGCAATTAACTT<br>TATTTGTACAATTATAAATATAATACCAAATAATATAAAATTAAATCAAATTCCACTTTTTCCATGATCAATTTT<br>AATTACAGCTATTTTATTAATCTTATCACTACCAGTTTTAGCTGGTGCTATTACAATACTATTAACTGATCGTA<br>ATTTAAATACATCATTTTTTGATCCAGCAGGAGGAGGAGATCCTATTTTATATCAACATTTATTC
AFNF025-12	*Metopolophium dirhodum*	AACTTTATACTTTTTATTTGGTATTTGATCAGGTATAATTGGATCATCTCTTAGAATTTTAATTCGTCTAGAAC<br>TAAGACAAATTAATTCTATTATTAATAATAATCAATTATACAATGTAATTGTAACAATTCATGCTTTTATTATA<br>ATTTTTTTTATAACAATACCAATTGTAATTGGTGGATTTGGAAATTGATTAATCCCTATAATAATAGGTTGCCC<br>TGATATATCATTCCCACGTTTAAATAACATTAGATTTTGATTATTACCCCCATCATTAATAATAATAATTTGTA<br>GTTTTTTAATTAATAATGGAACAGGAACAGGATGAACTATTTATCCACCTTTATCAAATAACATTGCACATAA<br>CAATATTTCAGTTGATTTAACTATTTTTTCATTACATTTAGCAGGAATCTCCTCAATTTTAGGAGCAATTAACT<br>TTATTTGTACAATTCTTAATATAATACCAAATAATATAAAATTAAATCAAATCCCTCTTTTCCCTTGATCAATTT<br>TAATTACAGCTATTTTATTAATTTTATCTTTACCAGTATTAGCTGGTGCTATTACAATATTATTAACTGATCGA<br>AATTTAAATACATCATTCTTTGATCCAGCAGGAGGAGGAGATCCTATTCTATATCAACATTTATTT
AFNF026-12	*Myzaphis rosarum*	AACTTTATATTTTTTATTTGGTATTTGATCAGGTTTAATTGGATCTTCTTTAAGAATTCTAATTCGATTAGAATT<br>AAGTCAAATTAATTCAATTATTAATAATAATCAATTATATAATGTAATTGTAACAATTCACGCTTTTATCATAA<br>TTTTTTTTATAACTATACCTATTGTAATTGGTGGATTTGGTAATTGATTAATCCCTTTAATAATAGGATGCCCT<br>GATATATCATTTCCACGATTAAATAATATTAGATTTTGATTATTACCCCCATCGCTAATAATAATAATCTGTAG<br>ATTCTTAATTAATAATGGAACAGGAACAGGATGAACTATTTATCCCCCACTATCAAACAACATTGCACATAAT<br>AATATTTCAGTCGATTTAACTATTTTTTCATTACATTTAGCAGGAATTTCCTCAATTCTAGGAGCAATTAATTTT<br>ATTTGCACAATTCTAAATATAATACCAAATAATTTAAAATTAAATCAAATTCCTCTATTCCCATGATCAATTTT<br>AATTACAGCCATTTTACTAATTTTATCATTACCAGTTCTAGCAGGTGCTATTACAATATTACTAACCGATCGAA<br>ATTTAAATACATCATTTTTTGATCCAGCAGGAGGAGGAGATCCTATTTTATATCAACATCTATTT
AFNF027-12	*Myzus persicae*	AACTTTATATTTTTTATTTGGTATTTGATCAGGTATAATTGGATCATCACTTAGAATCTTAATTCGTCTTGAATT<br>AAGACAAATTAATTCAATTATTAATAATAATCAATTATATAATGTTATTGTTACAATTCACGCTTTTATTATAA<br>TTTTTTTTATAACAATACCAATTGTTATTGGTGGATTTGGAAATTGGTTAATTCCTATAATAATAGGATGTCCT<br>GATATATCTTTCCCACGATTAAATAACATTAGATTCTGATTATTACCACCCTCATTAATAATAATAATTTGTAG<br>TTTTTTAATTAATAATGGAACAGGAACAGGATGAACTATTTACCCACCCTTATCAAATAATATTGCACATAAT<br>AATATTTCAGTTGATTTAACTATTTTTTCATTACATTTAGCAGGAATTTCATCAATTTTAGGAGCAATTAATTTT<br>ATTTGTACAATCTTAAATATAATACCAAACAATATAAAATTAAACCAAATCCCTTTATTTCCATGATCAATTTT<br>AATTACAGCTATTTTATTAATTTTATCTTTACCTGTTCTAGCAGGTGCTATTACAATATTATTAACTGATCGTAA<br>TTTAAATACTTCATTTTTTGACCCAGCAGGGGGAGGTGACCCAATCTTGTATCAACATTTATTT
AFNF<br>028-12	*Myzus padellus*	AACTTTATATTTTTTATTTGGTATTTGATCAGGTATAATTGGATCATCTCTTAGAATTTTAATTCGTCTTGAACT<br>AAGTCAAATTAATTCAATTATTAATAATAATCAATTATATAATGTAATTGTCACAATTCATGCCTTTATTATAA<br>TTTTTTTTATAACAATACCAATTGTGATTGGTGGTTTTGGAAATTGATTAATTCCTATAATAATAGGATGTCCT<br>GATATATCTTTTCCACGATTAAATAATATTAGTTTTTGATTATTACCACCATCATTAATAATAATAATTTGTAGT<br>TTTTTAATTAATAATGGAACAGGAACAGGATGAACTATTTATCCACCTTTATCAAATAATATTGCACATAATA<br>ATATTTCAGTTGATCTAACTATTTTTTCTCTACATTTAGCAGGAATTTCATCAATTCTAGGAGCAATTAATTTTA<br>TTTGCACAATTTTAAATATAATACCAAATAATATAAAATTAAATCAAATTCCTCTTTTCCCATGATCAATTTTAA<br>TTACAGCTATTTTATTAATTTTATCTTTACCTGTATTAGCAGGTGCAATTACAATATTATTAACTGATCGTAATT<br>TAAATACATCATTTTTTGATCCAGCAGGAGGAGGAGATCCTATTCTTTATCAACATTTATTC
AFNF029-12	*Nasonovia ribisnigri*	AACTTTATATTTTCTATTTGGTATTTGATCAGGTATAATTGGATCATCACTTAGAATTTTAATTCGTCTAGAAT<br>TAAGACAAATTAATTCAATTATTAATAATAATCAGTTATATAATGTAATTGTTACAATTCATGCTTTTATTATA<br>ATTTTTTTTATAACAATACCAATTGTTATTGGTGGTTTTGGAAACTGATTAATTCCTATAATAATAGGATGTCC<br>AGATATATCATTTCCACGTTTAAATAATATTAGATTTTGATTATTACCCCCCTCTTTAATAATAATAATTTGTAG<br>ATTCTTAATTAATAATGGAACAGGAACAGGATGAACTATTTATCCACCTTTATCAAATAATATTGCACATAAT<br>AATATTTCAGTTGATTTAACTATTTTTTCACTACATTTAGCAGGAATTTCATCAATCTTAGGAGCAATTAATTT<br>TATTTGTACAATTATAAATATAATACCAAACAATATAAAATTAAATCAAATTCCTTTATTTCCTTGATCAATTCT<br>AATTACAGCTATTTTATTAATTTTATCTTTACCAGTTTTAGCCGGTGCTATTACAATACTATTAACTGATCGTA<br>ACTTAAATACATCATTTTTTGATCCAGCAGGAGGAGGAGATCCTATTTTATATCAACATTTATTT
AFNF030-12	*Nasonovia pilosellae*	AACTTTATATTTTCTATTTGGTATTTGATCAGGTATAATTGGATCATCACTTAGAATTTTAATTCGTCTAGAAT<br>TAAGACAAATTAATTCAATTATTAATAATAATCAGTTATATAATGTAATTGTTACAATTCATGCTTTTATTATA<br>ATTTTTTTTATAACAATACCAATTGTTATTGGTGGTTTTGGAAACTGATTAATTCCTATAATAATAGGATGTCC<br>AGATATATCATTTCCACGTTTAAATAATATTAGATTTTGATTATTACCCCCCTCTTTAATAATAATAATTTGTAG<br>ATTCTTAATTAATAATGGAACAGGAACAGGATGAACTATTTATCCACCTTTATCAAATAATATTGCACATAAT<br>AATATTTCAGTTGATTTAACTATTTTTTCACTACATTTAGCAGGAATTTCATCAATCTTAGGAGCAATTAATTT<br>TATTTGTACAATTATAAATATAATACCAAACAATATAAAATTAAATCAAATTCCTTTATTTCCTTGATCAATTCT<br>AATTACAGCTATTTTATTAATTTTATCTTTACCAGTTTTAGCCGGTGCTATTACAATACTATTAACTGATCGTA<br>ACTTAAATACATCATTTTTTGATCCAGCAGGAGGAGGAGATCCTATTTTATATCAACATTTATTT

AFNF031-12	*Phorodon humuli*	AACTTTATATTTTTTATTTGGTATTTGATCAGGTATAATTGGATCATCACTTAGAATTTTAATTCGTCTTGAATT<br>AAGACAAATTAATTCAATTATTAATAACAATCAACTATATAATGTTATCGTTACAATTCATGCTTTTATTATAA<br>TTTTTTTTATAACAATACCAATTGTAATTGGTGGATTTGGAAATTGATTAATTCCTATAATAATAGGATGCCCT<br>GATATATCTTTCCCACGATTAAATAATATTAGATTCTGAATATTACCACCATCATTAATAATAATAATTTGTAG<br>TTTTTTAATTAACAATGGAACAGGAACAGGATGAACTATCTATCCACCCTTATCAAATAATATTGCACATAAT<br>AATATTTCAGTTGATTTAACTATTTTTTCACTTCATTTAGCAGGAATTTCATCAATTTTAGGAGCAATTAATTTT<br>ATTTGTACAATTTTAAACATAATACCAAATAATATAAAATTAAATCAAATTCCTCTTTTTCCATGATCAATTTTA<br>ATTACAGCTATTTTATTAATTTTATCTTTACCAGTTTTAGCAGGTGCTATTACAATATTATTAACTGATCGAAA<br>TTTAAATACATCATTTTTTGATCCAGCAGGAGGAGGGGATCCAATTTTATACCAACATTTATTT
AFNF032-12	*Rhopalomyzus lonicerae*|	AACTTTATATTTTTTATTTGGTATTTGATCAGGTATAATTGGATCATCTCTTAGAATTCTAATTCGATTAGAAC<br>TAAGACAAATTAATTCAATTATTAATAATAATCAATTATATAATGTAATTGTAACTATTCATGCTTTTATTATA<br>ATTTTTTTTATAACTATACCAATTGTAATTGGTGGATTTGGAAATTGATTAATTCCTATAATAATAGGATGCCC<br>TGATATATCTTTTCCACGATTAAATAATATTAGATTTTGACTATTACCACCATCATTAATAATAATAATTTGTA<br>GATTTTTAATTAATAATGGAACAGGAACAGGATGAACTATTTACCCACCATTATCAAACAATATTGCCCATAA<br>TAATATTTCAGTTGATTTAACTATTTTTTCATTACATTTAGCAGGAATCTCATCAATTTTAGGAGCAATTAATTT<br>TATCTGTACAATCTTAAATATAATACCAAATAACATAAAATTAAATCAAATTCCTTTATTTCCATGATCAATTTT<br>AATTACAGCAATCTTATTAATTTTATCTTTACCAGTATTAGCTGGTGCTATTACAATACTATTAACTGATCGTA<br>ATTTAAATACATCATTTTTTGATCCAGCAGGAGGAGGAGATCCTATTTTATATCAACATTTATTC
AFNF033-12	*Rhopalosiphum padi*	AACTTTATATTTTTTATTTGGTATTTGATCAGGTATAATTGGTTCATCCCTTAGAATCTTAATTCGATTAGAAC<br>TAAGTCAAATTAATTCAATTATTAATAATAATCAATTATATAATGTAATTGTTACAATTCACGCTTTTATTATA<br>ATTTTTTTTATAACTATACCAATTGTTATTGGTGGTTTTGGGAATTGACTAATTCCTATAATAATAGGATGCCC<br>TGATATATCATTTCCACGATTAAATAATATTAGATTTTGACTATTACCCCCTTCATTAATAATAATAATTTGTA<br>GTTTTATAATTAATAACGGAACAGGAACAGGATGAACAATTTATCCTCCTTTATCTAATAATATTGCTCATAA<br>TAATATTTCAGTTGATTTAACAATTTTTTCTCTACATTTAGCAGGAATCTCATCAATTTTAGGGGCAATTAATT<br>TTATTTGTACAATTTTAAATATAATACCTAATAATATAAAATTAAACCAAATTCCATTATTCCCTTGATCAATTT<br>TAATTACAGCTATATTATTAATTTTATCTTTACCTGTTTTAGCTGGTGCAATTACTATACTTCTTACTGATCGTA<br>ATTTAAATACATCATTCTTTGACCCTGCAGGAGGAGGAGATCCAATTCTTTATCAACATTTATTT
AFNF<br>034-12	*Sitobion avenae*	AACTCTATATTTCCTATTTGGTATTTGATCAGGTATAATTGGATCATCACTTAGAATTCTTATTCGTCTTGAAT<br>TAAGACAAATTAATTCAATTATTAATAATAATCAATTATATAATGTAATTGTTACAATCCATGCTTTTATTATA<br>ATTTTTTTTATAACTATACCAATTGTTATTGGTGGTTTTGGAAATTGATTAATTCCTATAATAATAGGATGTCC<br>TGATATATCATTTCCACGTTTAAATAATATTAGATTTTGATTACTACCACCATCATTAATAATAATAATCTGTA<br>GTTTCTTAATCAATAATGGAACAGGTACAGGATGAACTATTTACCCACCTTTATCAAATAATATTGCACATAA<br>TAATATTTCAGTTGATTTAACTATTTTTTCATTACATTTAGCAGGAATCTCATCAATTTTAGGAGCAATTAATTT<br>TATTTGTACAATTCTAAACATAATACCAAATAATATAAAATTAAATCAAATTCCTCTTTTCCCTTGATCAATTTT<br>AATTACAGCTATTTTATTAATTTTATCTTTACCAGTTTTAGCTGGTGCTATTACAATATTATTAACTGATCGAA<br>ATCTAAATACATCATTTTTTGATCCAGCAGGAGGAGGAGATCCAATTTTATATCAACATTTATTT
AFNF035-12	*Aphis gossypii*	AACTTTATATTTTTTATTTGGTATTTGATCAGGTATAATTGGTTCTTCTCTTAGAATTTTAATCCGATTAGAATT<br>AAGTCAAATTAATTCAATTATTAATAATAATCAATTATATAATGTAATTATTACAATTCATGCTTTTATTATAAT<br>TTTTTTTATAACTATACCAATCGTTATTGGAGGTTTTGGAAATTGATTAATTCCTATAATAATAGGATGTCCAG<br>ATATATCTTTTCCACGACTAAATAATATTAGATTCTGATTATTACCACCCTCATTAATAATAATAATTTGCAGA<br>TTTATAATTAATAACGGAACAGGAACAGGATGAACTATTTATCCACCTTTATCAAATAATATTGCTCATAATA<br>ATATTTCAGTAGACTTAACTATTTTTTCCCTACATTTAGCAGGTATCTCATCAATTTTAGGAGCAATTAATTTC<br>ATCTGTACTATCTTAAATATAATACCTAATAATATAAAATTAAATCAAATTCCTCTATTTCCATGATCAATTTTA<br>ATTACAGCTATATTATTAATTTTATCCTTACCTGTATTAGCTGGTGCTATTACTATATTATTAACAGATCGAAA<br>TTTAAATACATCATTTTTTGATCCAGCAGGTGGGGGAGACCCTATTCTTTATCAACATTTATTT
AFNF036-12	*Aphis gossypii*	AACTTTATATTTTTTATTTGGTATTTGATCAGGTATAATTGGTTCTTCTCTTAGAATTTTAATCCGATTAGAATT<br>AAGTCAAATTAATTCAATTATTAATAATAATCAATTATATAATGTAATTATTACAATTCATGCTTTTATTATAAT<br>TTTTTTTATAACTATACCAATCGTTATTGGAGGTTTTGGAAATTGATTAATTCCTATAATAATAGGATGTCCAG<br>ATATATCTTTTCCACGACTAAATAATATTAGATTCTGATTATTACCACCCTCATTAATAATAATAATTTGCAGA<br>TTTATAATTAATAACGGAACAGGAACAGGATGAACTATTTATCCACCTTTATCAAATAATATTGCTCATAATA<br>ATATTTCAGTAGACTTAACTATTTTTTCCCTACATTTAGCAGGTATCTCATCAATTTTAGGAGCAATTAATTTC<br>ATCTGTACTATCTTAAATATAATACCTAATAATATAAAATTAAATCAAATTCCTCTATTTCCATGATCAATTTTA<br>ATTACAGCTATATTATTAATTTTATCCTTACCTGTATTAGCTGGTGCTATTACTATATTATTAACAGATCGAAA<br>TTTAAATACATCATTTTTTGATCCAGCAGGTGGGGGAGACCCTATTCTTTATCAACATTTATTT
AFNF037-12	*Aphis gossypii*	AACTTTATATTTTTTATTTGGTATTTGATCAGGTATAATTGGTTCTTCTCTTAGAATTTTAATCCGATTAGAATT<br>AAGTCAAATTAATTCAATTATTAATAATAATCAATTATATAATGTAATTATTACAATTCATGCTTTTATTATAAT<br>TTTTTTTATAACTATACCAATCGTTATTGGAGGTTTTGGAAATTGATTAATTCCTATAATAATAGGATGTCCAG<br>ATATATCTTTTCCACGACTAAATAATATTAGATTCTGATTATTACCACCCTCATTAATAATAATAATTTGCAGA<br>TTTATAATTAATAACGGAACAGGAACAGGATGAACTATTTATCCACCTTTATCAAATAATATTGCTCATAATA<br>ATATTTCAGTAGACTTAACTATTTTTTCCCTACATTTAGCAGGTATCTCATCAATTTTAGGAGCAATTAATTTC<br>ATCTGTACTATCTTAAATATAATACCTAATAATATAAAATTAAATCAAATTCCTCTATTTCCATGATCAATTTTA<br>ATTACAGCTATATTATTAATTTTATCCTTACCTGTATTAGCTGGTGCTATTACTATATTATTAACAGATCGAAA<br>TTTAAATACATCATTTTTTGATCCAGCAGGTGGGGGAGACCCTATTCTTTATCAACATTTATTT
AFNF038-12	*Aphis gossypii*	AACTTTATATTTTTTATTTGGTATTTGATCAGGTATAATTGGTTCTTCTCTTAGAATTTTAATCCGATTAGAATT<br>AAGTCAAATTAATTCAATTATTAATAATAATCAATTATATAATGTAATTATTACAATTCATGCTTTTATTATAAT<br>TTTTTTTATAACTATACCAATCGTTATTGGAGGTTTTGGAAATTGATTAATTCCTATAATAATAGGATGTCCAG<br>ATATATCTTTTCCACGACTAAATAATATTAGATTCTGATTATTACCACCCTCATTAATAATAATAATTTGCAGA<br>TTTATAATTAATAACGGAACAGGAACAGGATGAACTATTTATCCACCTTTATCAAATAATATTGCTCATAATA<br>ATATTTCAGTAGACTTAACTATTTTTTCCCTACATTTAGCAGGTATCTCATCAATTTTAGGAGCAATTAATTTC<br>ATCTGTACTATCTTAAATATAATACCTAATAATATAAAATTAAATCAAATTCCTCTATTTCCATGATCAATTTTA<br>ATTACAGCTATATTATTAATTTTATCCTTACCTGTATTAGCTGGTGCTATTACTATATTATTAACAGATCGAAA<br>TTTAAATACATCATTTTTTGATCCAGCAGGTGGGGGAGACCCTATTCTTTATCAACATTTATTT

AFNF039-12	*Aphis gossypii*	AACTTTATATTTTTTATTTGGTATTTGATCAGGTATAATTGGTTCTTCTCTTAGAATTTTAATCCGATTAGAATT<br>AAGTCAAATTAATTCAATTATTAATAATAATCAATTATATAATGTAATTATTACAATTCATGCTTTTATTATAAT<br>TTTTTTTATAACTATACCAATCGTTATTGGAGGTTTTGGAAATTGATTAATTCCTATAATAATAGGATGTCCAG<br>ATATATCTTTTCCACGACTAAATAATATTAGATTCTGATTATTACCACCCTCATTAATAATAATAATTTGCAGA<br>TTTATAATTAATAACGGAACAGGAACAGGATGAACTATTTATCCACCTTTATCAAATAATATTGCTCATAATA<br>ATATTTCAGTAGACTTAACTATTTTTTCCCTACATTTAGCAGGTATCTCATCAATTTTAGGAGCAATTAATTTC<br>ATCTGTACTATCTTAAATATAATACCTAATAATATAAAATTAAATCAAATTCCTCTATTTCCATGATCAATTTTA<br>ATTACAGCTATATTATTAATTTTATCCTTACCTGTATTAGCTGGTGCTATTACTATATTATTAACAGATCGAAA<br>TTTAAATACATCATTTTTTGATCCAGCAGGTGGGGGAGACCCTATTCTTTATCAACATTTATTT
